# Pregnane X receptor reduces particulate matter-induced type 17 inflammation in atopic dermatitis

**DOI:** 10.3389/fimmu.2024.1415350

**Published:** 2024-09-27

**Authors:** Ji Su Lee, Youngae Lee, Sunhyae Jang, Jang-Hee Oh, Dong Hun Lee, Soyun Cho

**Affiliations:** ^1^ Department of Dermatology, Seoul National University College of Medicine, Seoul, Republic of Korea; ^2^ Department of Dermatology, Seoul National University Hospital, Seoul, Republic of Korea; ^3^ Laboratory of Cutaneous Aging Research, Biomedical Research Institute, Seoul National University Hospital, Seoul, Republic of Korea; ^4^ Institute of Human-Environment Interface Biology, Medical Research Center, Seoul National University, Seoul, Republic of Korea; ^5^ Laboratory of Cutaneous Aging and Hair Research, Clinical Research Institute, Seoul National University Hospital, Seoul, Republic of Korea; ^6^ Department of Dermatology, Seoul Metropolitan Government – Seoul National University (SMG-SNU) Boramae Medical Center, Seoul, Republic of Korea

**Keywords:** particulate matter, air pollution, pregnane X receptor, atopic dermatitis, type 17 inflammation

## Abstract

**Background:**

Epidemiological evidence suggests that particulate matter (PM) exposure can trigger or worsen atopic dermatitis (AD); however, the underlying mechanisms remain unclear. Recently, pregnane X receptor (PXR), a xenobiotic receptor, was reported to be related to skin inflammation in AD.

**Objectives:**

This study aimed to explore the effects of PM on AD and investigate the role of PXR in PM-exposed AD.

**Methods:**

*In vivo* and *in vitro* AD-like models were employed, using BALB/c mice, immortalized human keratinocytes (HaCaT), and mouse CD4 ***^+^*** T cells.

**Results:**

Topical application of PM significantly increased dermatitis score and skin thickness in AD-like mice. PM treatment increased the mRNA and protein levels of type 17 inflammatory mediators, including interleukin (IL)-17A, IL-23A, IL-1β, and IL-6, in AD-like mice and human keratinocytes. PM also activated PXR signaling, and PXR knockdown exacerbated PM-induced type 17 inflammation in human keratinocytes and mouse CD4 ***^+^*** T cells. In contrast, PXR activation by rifampicin (a human PXR agonist) reduced PM-induced type 17 inflammation. Mechanistically, PXR activation led to a pronounced inhibition of the nuclear factor kappa B (NF-κB) pathway.

**Conclusion:**

In summary, PM exposure induces type 17 inflammation and PXR activation in AD. PXR activation reduces PM-induced type 17 inflammation by suppressing the NF-κB signaling pathway. Thus, PXR represents a promising therapeutic target for controlling the PM-induced AD aggravation.

## 1 Introduction

Particulate matter (PM) from ambient air is a key component of air pollutants and is a complex mixture of solid and liquid particles, including nitrates, sulfates, elemental and organic carbon, organic compounds (e.g., polycyclic aromatic hydrocarbons (PAHs)), biological compounds (e.g., endotoxins and cell fragments), and metals (e.g., iron, copper, nickel, and zinc) (1). PM can be classified according to particle diameter as follows: PM_10_ (coarse particles, ≤10 μm), PM_2.5_ (fine particles, ≤2.5 μm), and PM_0.1_ (ultrafine particles, ≤0.1 μm) (1, 2). Most studies on the effects of PM on health have focused on cardiovascular and respiratory diseases (3); however, increasing evidence demonstrates that PM also detrimentally affects the skin (2, 4–7).

Atopic dermatitis (AD) is a chronic, relapsing, inflammatory skin disease that places a significant burden on healthcare resources and patients’ quality of life (8). AD is a multifactorial disease resulting from complex interactions among genetic predisposition, environmental factors, immune dysregulation, and skin barrier dysfunction. The prevalence of AD is continuously increasing, particularly in countries with rapidly developing urban areas, which emphasizes the role of the environment in AD pathogenesis (8–10). Several epidemiological studies have indicated that PM exposure contributes to the aggravation and development of AD (2, 6, 11–14). However, these studies were primarily focused on epidemiological perspectives and did not investigate the mechanisms underlying the effects of PM on AD, which remain unclear.

One of the putative mechanisms involved is xenobiotic metabolism, which is the process of detoxifying exogenous (e.g., pollutants) and endogenous (e.g., bilirubin) chemicals (15–17). This process is regulated by xenobiotic receptors, including pregnane X receptor (PXR), aryl hydrocarbon receptor (AHR), and constitutive androstane receptor (CAR). Xenobiotic receptors function beyond xenobiotic metabolism and are additionally involved in various cellular processes, including inflammation, oxidative stress, cell proliferation and death, lipid metabolism, tissue injury and repair, and cancer development (15). As the first barrier of the human body, the skin is exposed to numerous factors, such as pollutants, that can serve as ligands and activate xenobiotic receptors (18) which are expressed in epidermal keratinocytes, dermal fibroblasts, and immune cells (15). Some studies have shown that xenobiotic metabolism is triggered in the skin of patients with AD (15–18). However, most studies have focused on AHR, and little is known about the role of PXR in PM-exposed AD skin. Interestingly, PXR has demonstrated both anti-inflammatory (19–22) and pro-inflammatory effects (18, 23), suggesting context- and ligand-dependent dual roles (15). Therefore, additional studies are needed to determine the specific function of PXR in PM-exposed AD.

In the present study, we aimed to elucidate PM-induced effects on AD and the role of PXR in PM-exposed AD *in vivo* in mice skin and *in vitro* in human cell lines. We observed that PM induces type 17 inflammation and PXR activation. Activated PXR reduces PM-induced type 17 inflammatory mediators through suppression of the nuclear factor kappa B (NF-κB) signaling pathway in AD. Thus, targeting PXR is a promising strategy to attenuate the PM-induced flare-up or progression of AD. Our results provide novel insights into protecting skin health against air pollution.

## 2 Materials and methods

Detailed experimental methods are presented in the **Supplementary Methods**.

### 2.1 PM preparation

Standard reference material (SRM) 2786 (PM) was purchased from the National Institute of Standards and Technology (NIST, Gaithersburg, MD, USA). This material, with a mean particle diameter of 2.8 μm, was collected from ambient air in Prague, Czechia and represents atmospheric PM_2.5_ obtained from the urban environment containing PAH, metals, biological combustion products, and other substances (**Supplementary Table 1**) (24). SRM 2786 was resuspended in phosphate-buffered saline (PBS) at a concentration of 50 mg/ml and stored at 4°C until use. SRM stock solution was vortexed for 5 min at maximum speed and sonicated for 5 min using a Bandelin sonicator (Bandelin, Berlin, Germany) immediately before treatment.

### 2.2 Animals, induction of AD, and PM treatment

**Figure 1:**
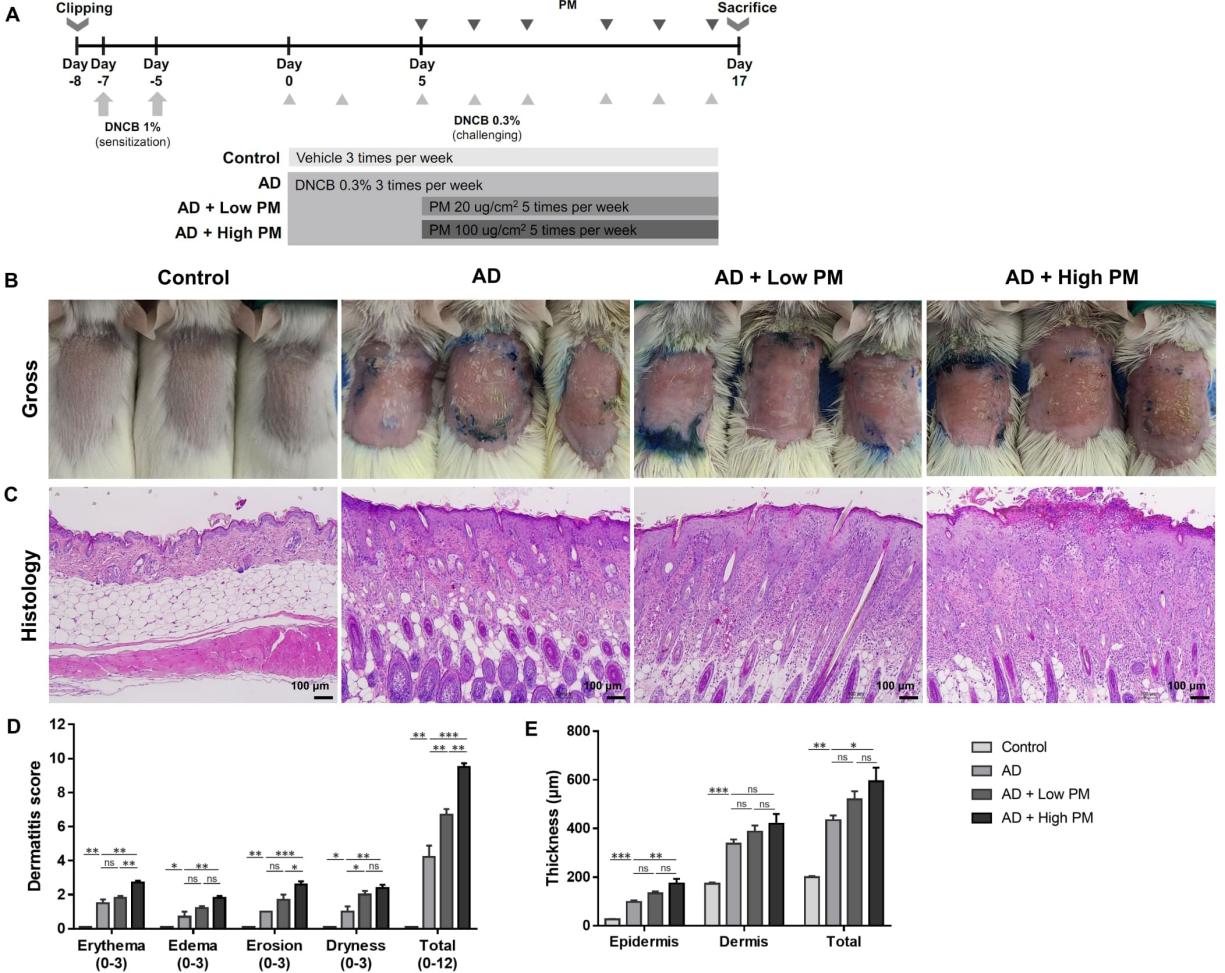
Particulate matter (PM) exposure aggravates atopic dermatitis (AD)-like features in AD-like mice in clinical and histopathological examinations. **(A)** Mouse experimental schedule. We generated a 2,4-dinitrochlorobenzene (DNCB)-induced AD-like model using BALB/c mice. AD-like mice were additionally treated with either low (20 µg/cm^2^) or high (100 µg/cm^2^) concentration of PM. **(B, D)** DNCB-treated mice showed AD-like skin lesions with erythema, edema, erosion, and dryness/scales. Dermatitis score increased in the AD group and further increased in the AD + PM groups in a dose-dependent manner. **(C, E)** Histology of the PM-exposed AD-like mice stained with hematoxylin and eosin. DNCB-treated mice showed AD-like features such as hyperkeratosis, acanthosis, spongiosis, and inflammatory cell infiltration. PM further increased the skin thickness. Data are representative of two independent experiments and are shown as the mean ± SEM (n = 5 mice in each group). ns, nonsignificant; *P <.05; **P <.01; ***P <.001. P-values were obtained by the unpaired Student’s *t* test and one-way ANOVA.

We used the 2,4-dinitrochlorobenzene (DNCB)-induced AD-like mouse model, as previously described (25–30). Six-week-old female BALB/c mice were purchased from Orient Bio Inc. (Seongnam, Gyeonggi, Republic of Korea) and housed under semi-specific pathogen-free conditions with individually ventilated cages (24 ± 2 °C with a 12-h light-dark cycle). They were fed standard laboratory chow and water *ad libitum*. The experimental schedule is summarized in **Figure 1A**. After 1 week of acclimation, the dorsal area of the mice was shaved and depilated (1.5 × 2 cm^2^). On Day 7 and Day 5, after shaving and depilation, 100 µL of 1% DNCB dissolved in an acetone: olive oil mixture (3:1 vol/vol) was applied to the dorsal skin of the mice (DNCB sensitization). The cutaneous DNCB-sensitized mice were divided into four groups: (1) Control, (2) AD, (3) AD + Low PM, and (4) AD + High PM. From Day 0 to Day 16, the vehicle (acetone and olive oil mixture) was applied to the dorsum three times a week in the control group. In AD, AD + low PM, and AD + high PM, 200 µL of 0.3% DNCB was applied to the dorsum three times a week (DNCB challenge). From Day 5, 20 µg/cm^2^ and 100 µg/cm^2^ of PM (SRM 2786) in PBS (31, 32) was additionally applied 1 h after DNCB application in the AD + low PM and AD + high PM group, respectively.

### 2.3 Cell culture, induction of AD, and PM treatment

HaCaT cells (immortalized human keratinocytes) were cultured in Dulbecco’s modified Eagle’s medium (DMEM, Welgene, Daegu, Republic of Korea) supplemented with 10% fetal bovine serum (FBS, Thermo Fisher Scientific, Waltham, MA, USA) and 1% penicillin-streptomycin at 37°C in a humidified atmosphere of 5% CO_2_. Cells were first seeded (1.5 × 10^5^ cells/dish in a 35 mm dish) and grown for 2–3 days and then starved with 0% FBS-DMEM for 1 day at >80% confluence. Subsequently, cells were co-stimulated with tumor necrosis factor alpha (TNF-α) (2 ng/mL) and interferon gamma (IFN-γ) (10 ng/mL) to induce AD-like keratinocytes as previously described (33–37). TNF-α and IFN-γ-treated AD-like keratinocytes are known to produce type 2 inflammation-mediated chemokines like thymus and activation-regulated chemokine (TARC) and macrophage-derived chemokine (MDC) and decrease the expression of barrier-related proteins including filaggrin and loricrin (33–37). Recombinant TNF-α and IFN-γ were purchased from R&D Systems (Minneapolis, MN, USA). After 1 h, cells were additionally treated with PM (100 μg/ml). Thereafter, the cells were harvested for mRNA or protein analysis at 4 h after treatment, and supernatants were harvested for protein analysis at 24 h after treatment.

### 2.4 Isolation and activation of mouse CD4^+^ T cells

Mouse CD4^+^ T cells from the spleen were isolated using magnetic activated cell sorting (MACS) LS columns, adhering to the manufacturer’s guidelines (Miltenyi Biotec, Bergisch Gladbach, North Rhine-Westphalia, Germany). For the activation of mouse T cells, purified CD4^+^ T cells (2 × 10^5^) were stimulated with plate-bound anti-CD3ϵ (5 µg/ml) and soluble anti-CD28 (2 µg/ml) antibodies for 72 h, either in the presence of a vehicle or PM. CD4^+^ T cells were cultured in RPMI 1640 (Welgene) supplemented with HEPES, non-essential amino acids, sodium pyruvate, glutamine, penicillin-streptomycin, and 10% FBS. Antibodies against CD3ϵ (clone 145-2C11) and CD28 (clone 37.51) were purchased from eBioscience (San Diego, CA, USA). Mouse CD4 MicroBeads (clone L3T4) were purchased from Miltenyi Biotec. All culture reagents for mouse CD4^+^ T cells were purchased from Sigma-Aldrich (St. Louis, MO, USA). Mouse interleukin (IL)-4 and IL-17A production levels in the culture supernatants were measured using ELISA, following the manufacturer’s protocol from BioLegend (San Diego, CA, USA).

### 2.5 Statistics

All statistical analyses were performed using SPSS 19.0 software (IBM, Armonk, NY, USA). The results are expressed as the mean ± standard error of means (SEM). Comparisons between two groups were performed using unpaired Student’s *t*-test. Comparisons between three or more groups were performed using one-way analysis of variance (ANOVA) with Tukey’s *post hoc* test. Data are representative of two to three independent experiments (n = 3–5 per group). P-values <.05 indicated significance.

### 2.6 Study approval

The animal experimental protocol was approved by the Seoul National University Hospital Institutional Animal Care and Use Committee (No.19-0090-S1A0). All experiments were performed in accordance with the approved experimental protocol.

## 3 Results

### 3.1 PM exposure aggravates AD-like inflammation

We employed the DNCB-induced AD-like mouse model, which is a well-established AD-like *in vivo* model (25–30). DNCB-treated mice showed AD-like skin lesions characterized by erythema, edema, erosion, and dryness/scales (**Figure 1B**). Histological examination revealed hyperkeratosis, acanthosis, spongiosis, and extensive infiltration of inflammatory cells in the epidermis and dermis (**Figure 1C**). Topical application of low (20 µg/cm^2^) or high (100 µg/cm^2^) concentrations of PM_2.5_ (SRM 2786) further aggravated the AD-like skin lesions (**Figures 1B, C**). In addition to individual scores reflecting erythema, edema, erosion, and dryness, the overall dermatitis score was significantly increased in the AD group compared to that in the control group and further increased in the PM groups in a dose-dependent manner (AD, 4.20 ± 0.68; AD + low PM, 6.70 ± 0.34; AD + high PM, 9.5 ± 0.22) (**Figure 1D**). Similarly, epidermal thickness and skin thickness (sum of epidermal and dermal thicknesses) were significantly increased in the AD group and further increased in the high-PM groups (**Figure 1E**).

### 3.2 PM exposure increases type 17 inflammation in AD-like inflammation

**Figure 2:**
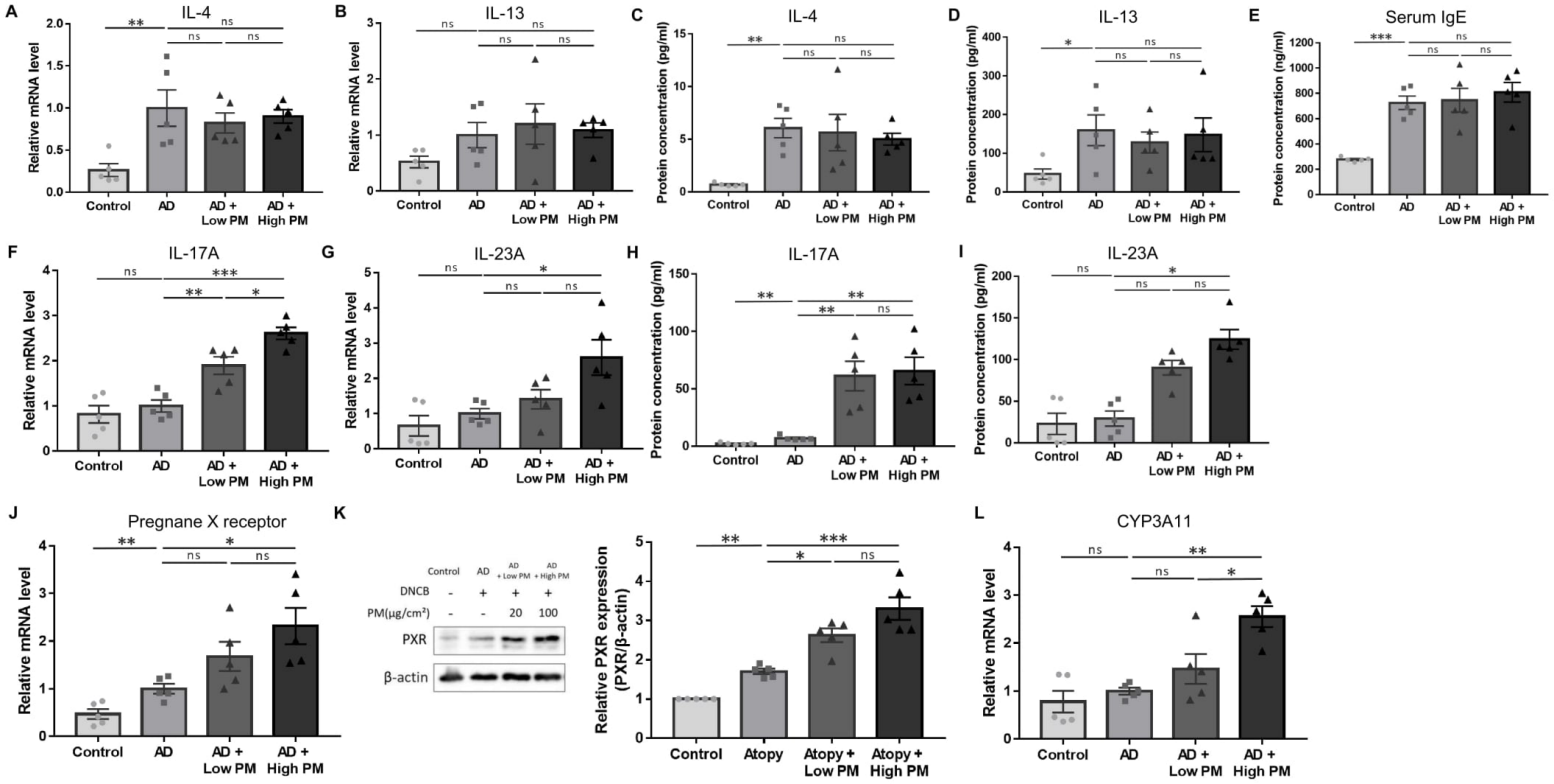
Change in the expression of type 2 and type 17 cytokines, pregnane X receptor (PXR), and downstream genes after atopic dermatitis (AD) induction and additional particulate matter (PM) treatment in mice. **(A, B)** mRNA and **(C, D)** protein levels of type 2 cytokines (interleukin (IL)-4 and IL-13) and **(E)** serum immunoglobulin E levels increased after AD induction but did not further increase after PM treatment. **(F, G)** mRNA and **(H, I)** protein levels of type 17 cytokines (IL-17A and IL-23A) increased slightly after AD induction and further increased after PM treatment. **(J)** mRNA and **(K)** protein levels of PXR increased after additional PM treatment. **(L)** Downstream gene of PXR in mice (*CYP3A11*) increased after additional PM treatment. Data are representative of two independent experiments and are shown as the mean ± SEM (n = 5 mice in each group). The mRNA data were normalized to the AD group. ns, nonsignificant; *P <.05; **P <.01; ***P <.001. P-values were obtained using the unpaired Student’s t test and one-way ANOVA.

Next, we analyzed key pathogenic cytokines associated with AD in skin tissues. As expected, the mRNA (**Figures 2A, B**) and protein (**Figures 2C, D**) levels of type 2 cytokines, including IL-4 and IL-13, were increased after AD induction. Serum immunoglobulin E (IgE) levels were also significantly increased in AD-like mice skin lesions (**Figure 2E**). However, IL-4, IL-13, and serum IgE levels did not show a further significant increase by additional PM treatment (**Figures 2A–E**).

Previous studies have suggested that in addition to the type 2 immune response, type 17 immune response also contributes to AD pathogenesis (38–41) and is associated with PM exposure (17, 42–48). We observed a modest increase in the levels of major type 17 inflammatory mediators, IL-17A and IL-23A, after AD induction (**Figures 2F–I**). Notably, IL-17A mRNA and protein were significantly increased with both low and high PM exposure (**Figures 2F, H**), and those of IL-23A were significantly increased after high PM treatment (**Figures 2G, I**). Other type 17 inflammation-related cytokines including IL-1β and IL-6 were increased after AD induction and showed a further incremental tendency after additional PM treatment (**Supplementary Figure 1**).

### 3.3 PM exposure activates PXR in AD-like inflammation

To investigate the role of PXR in PM-exposed AD, we examined the expression of PXR and its downstream genes in the skin. Indeed, the levels of PXR mRNA and protein were significantly increased in the AD group and were further increased with additional PM treatment, particularly for high PM exposure (**Figures 2J, K**). Consistently, high PM exposure significantly induced downstream PXR genes in mice such as *CYP3A11* (**Figure 2L**), indicating that PM exposure activated the PXR signaling pathway.

### 3.4 PM exposure increases type 17 inflammation-related cytokine production and activates PXR in AD-like keratinocytes

**Figure 3:**
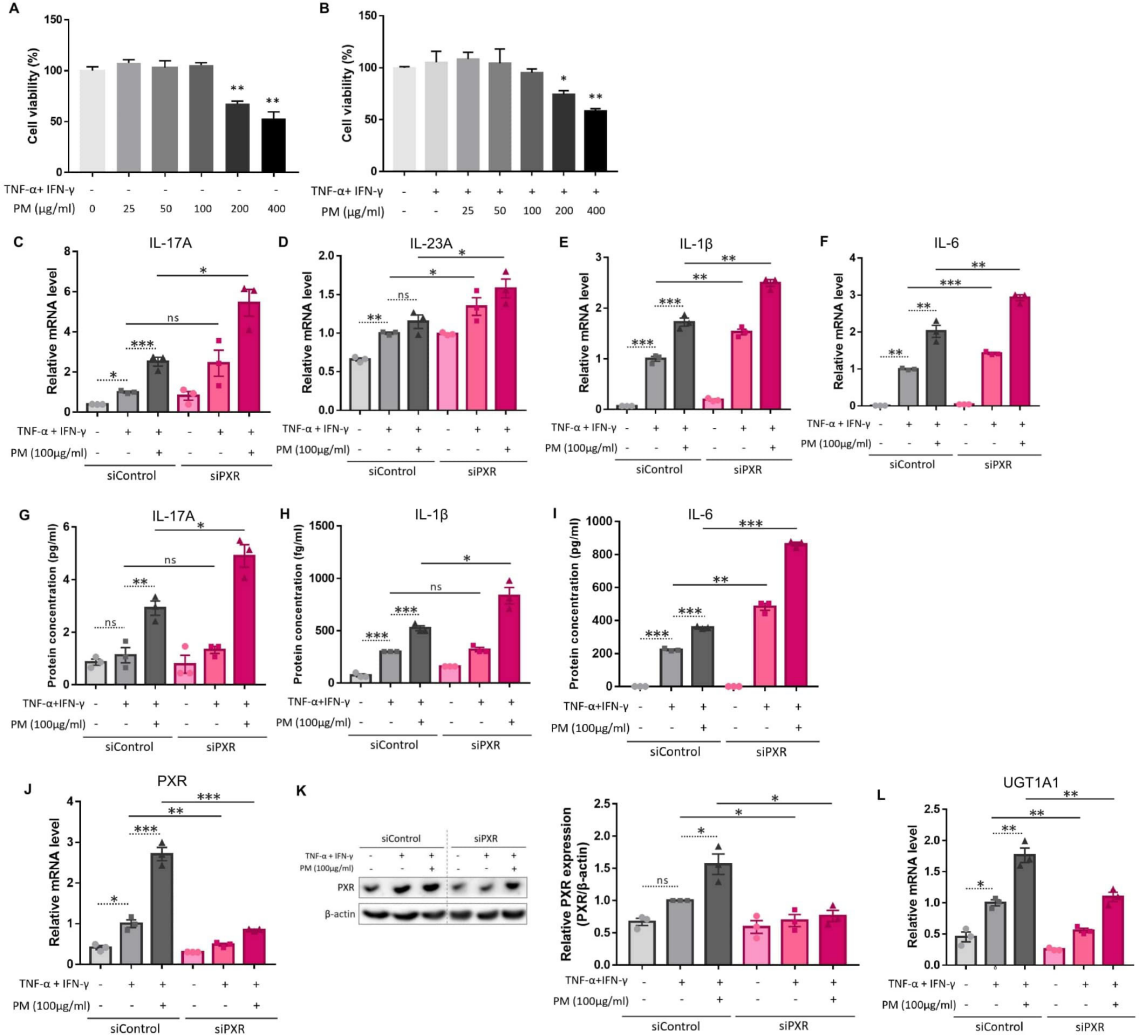
Comparison of the expression of type 17 inflammation-related cytokines, pregnane X receptor (PXR), and its downstream gene after atopic dermatitis (AD) induction and additional particulate matter (PM) treatment between control and PXR siRNA-transfected keratinocytes. The cell viability of **(A)** normal keratinocytes and **(B)** AD-like keratinocytes (TNF-α/IFN-γ-treated keratinocytes) was sustained with PM concentration increment until 100 μg/ml, after which it started to decrease. **(C–F)** mRNA and **(G–I)** protein levels of type 17 inflammatory cytokines (IL-17A, IL-23A, IL-1β, and IL-6) increased after AD induction, and further increased after PM treatment. IL-23A protein was undetectable. **(C–I)** Increased type 17 inflammation was exaggerated after PXR gene silencing using siRNA transfection. **(J)** mRNA and **(K)** protein levels of PXR increased after additional PM treatment. **(L)** Downstream gene of PXR in humans (*UGT1A1*) increased after additional PM treatment. **(J–L)** PM-induced PXR activation was suppressed after PXR siRNA transfection. Data are representative of three independent experiments and are shown as the mean ± SEM (n = 3 in each group). The mRNA data were normalized to the AD-like keratinocytes transfected with control siRNA without PM treatment. ns, nonsignificant; *P <.05; **P <.01; ***P <.001. P-values were obtained using the unpaired Student’s t test and one-way ANOVA.

We employed a TNF-α- and IFN-γ-stimulated *in vitro* AD-like HaCaT cell model, which produces type 2 inflammation-mediated chemokines and decreases the expression of barrier-related proteins, as previously reported (33–37). We further administered 100 μg/ml of PM to these cells, which was the highest concentration that did not induce cytotoxicity (**Figures 3A, B**). Similar to AD-like inflammation in mice, AD-like keratinocytes showed an increase in type 17 inflammation-related cytokines (IL-17A, IL-23A, IL-1β, and IL-6), which was further significantly increased after additional PM treatment, except for IL-23A (**Figures 3C–I**), whose protein was undetectable. Specifically, IL-17A mRNA and protein levels were considerably increased after PM treatment (**Figures 3C, G**), consistent with *in vivo* findings (**Figures 2F, H**). Moreover, PM treatment in keratinocytes significantly increased PXR mRNA levels and its downstream gene in humans, *UGT1A1* (**Figures 3J, L**). Luciferase activity of PXR increased in the PM-treated AD-like keratinocytes (**Supplementary Figure 2A**). PXR protein levels were also increased in PM-exposed AD-like keratinocytes (**Figure 3K**). Collectively, the PM-treated AD-like keratinocytes recapitulated the characteristic features of PM-treated AD-like mice, such as type 17 inflammation and PXR activation.

### 3.5 PXR activation reduces PM-induced type 17 inflammation

**Figure 4:**
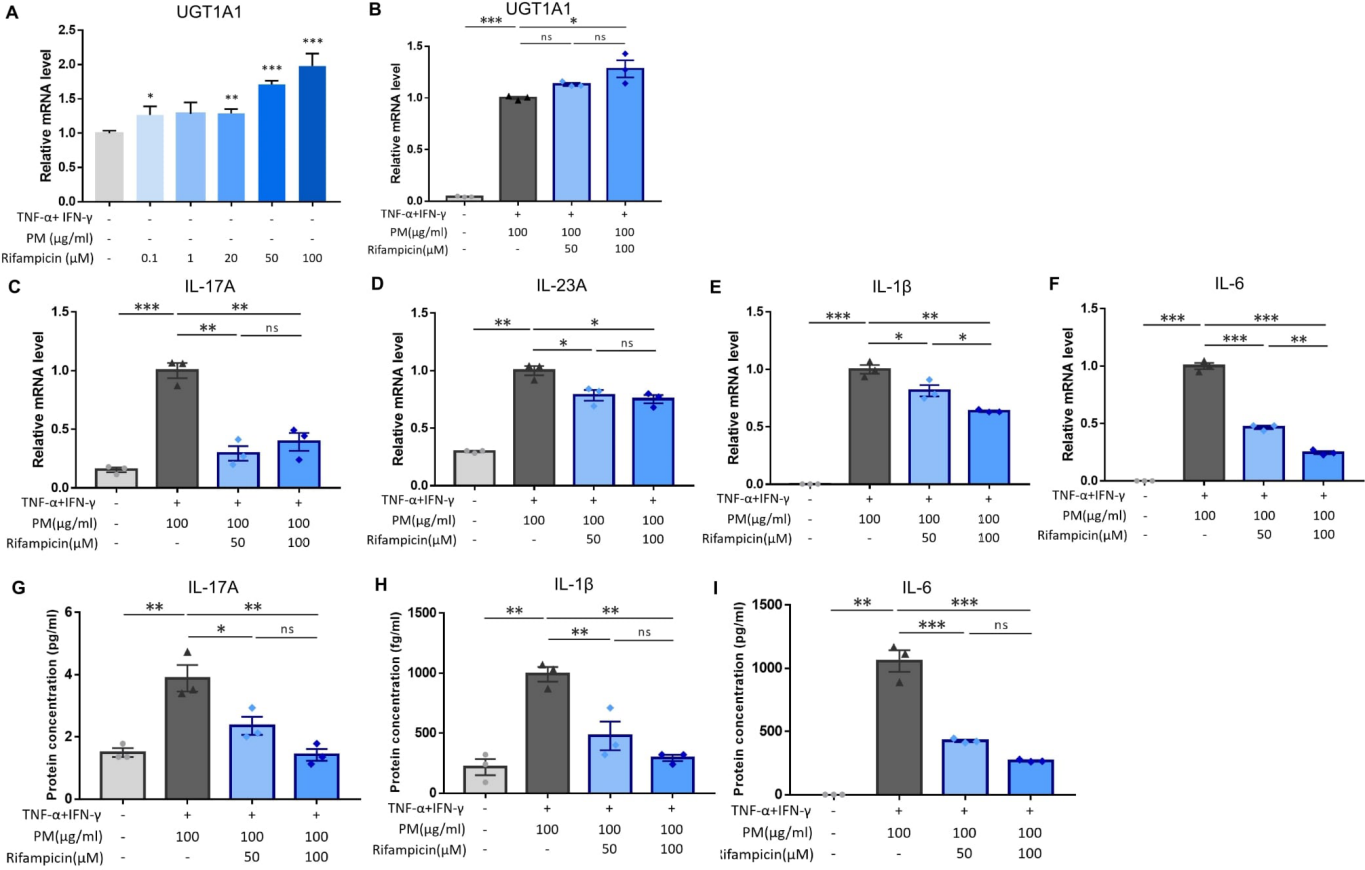
Influence of rifampicin (human pregnane X receptor (PXR) agonist) treatment in particulate matter (PM)-induced type 17 inflammation in atopic dermatitis (AD). **(A)** *UGT1A1*, a downstream gene of human PXR, increased by rifampicin treatment in a dose-dependent manner. **(B)** Rifampicin further increased the mRNA level of *UGT1A1* after PM treatment. **(C–F)** mRNA and **(G–I)** protein levels of type 17 inflammation-related cytokines (IL-17A, IL-23A, IL-1β, and IL-6) increased by PM treatment in AD-like keratinocytes, and rifampicin reduced the effects of PM in a dose-dependent manner. Data are representative of three independent experiments and are shown as the mean ± SEM (n = 3 in each group). For **(A, B)**, the mRNA data were normalized to the untreated keratinocytes, while for **(C–I)**, the mRNA data were normalized to the PM-treated AD-like keratinocytes without rifampicin treatment. ns, nonsignificant; *P <.05; **P <.01; ***P <.001. P-values were obtained using the unpaired Student’s t test and one-way ANOVA.

Previous studies have demonstrated that PXR suppresses inflammation and regulates tissue damage (19–22). However, other reports suggest that PXR activation triggers inflammatory responses in a context-specific manner (15, 18, 23). We knocked down PXR in keratinocytes using PXR siRNA prior to AD induction and PM treatment to clarify the specific role of PXR in PM-induced type 17 inflammation in AD. The mRNA and protein levels of PXR were decreased after PXR siRNA transfection (**Figures 3J, K**), along with its downstream gene, *UGT1A1* (**Figure 3L**). Compared to control siRNA-transfected cells, PXR siRNA-transfected keratinocytes showed a significant increase in the mRNA levels of type 17 inflammation-related cytokines (IL-17A, IL-23A, IL-1β, and IL-6) after PM treatment (**Figures 3C–F**). Furthermore, the increase in IL-17A, IL-1β, and IL-6 was confirmed at the protein level (**Figures 3G–I**). Consistently, SPA70, a human PXR antagonist, increased the mRNA levels of IL-1β, IL-6, and IL-23A in PM-treated AD-like keratinocytes (**Supplementary Figures 3A–C**). In the meanwhile, CH223191, an AHR antagonist, increased the mRNA levels of IL-1β, IL-6, and IL-23A in PM-treated AD-like keratinocytes (**Supplementary Figures 4A–C**). Our findings suggest that PXR activation induced by PM exposure limits the PM-induced type 17 inflammation in AD, aside from AHR. To confirm these assumptions, we treated keratinocytes with rifampicin, a potent human PXR agonist (15) before AD induction and PM treatment. Rifampicin increased the luciferase activity of PXR (**Supplementary Figure 2B**). Rifampicin increased the mRNA level of *UGT1A1* in a dose-dependent manner (**Figure 4A**) and accelerated the PM-induced increase in *UGT1A1* in AD-like keratinocytes (**Figure 4B**), indicating the activation of the PXR signaling pathway. Indeed, rifampicin treatment reduced PM-induced type 17 inflammation in AD-like keratinocytes, with decreased mRNA (IL-17A, IL-23A, IL-1β, and IL-6) and protein (IL-17A, IL-1β, and IL-6) levels of type 17 cytokines after PM treatment in rifampicin-treated keratinocytes (**Figures 4C–I**). When comparing the effect of rifampicin treatment, the expressions of IL-1β and IL-6 were higher in PXR siRNA-transfected AD-like keratinocytes compared to control siRNA-transfected AD-like keratinocytes (**Supplementary Figures 5A, B**). This finding suggests that the effect of rifampicin was partially canceled by PXR knockdown.

### 3.6 Exposure to PM amplifies IL-17A production in mouse CD4^+^ T cells, with a more pronounced increase observed in PXR knockdown conditions

**Figure 5:**
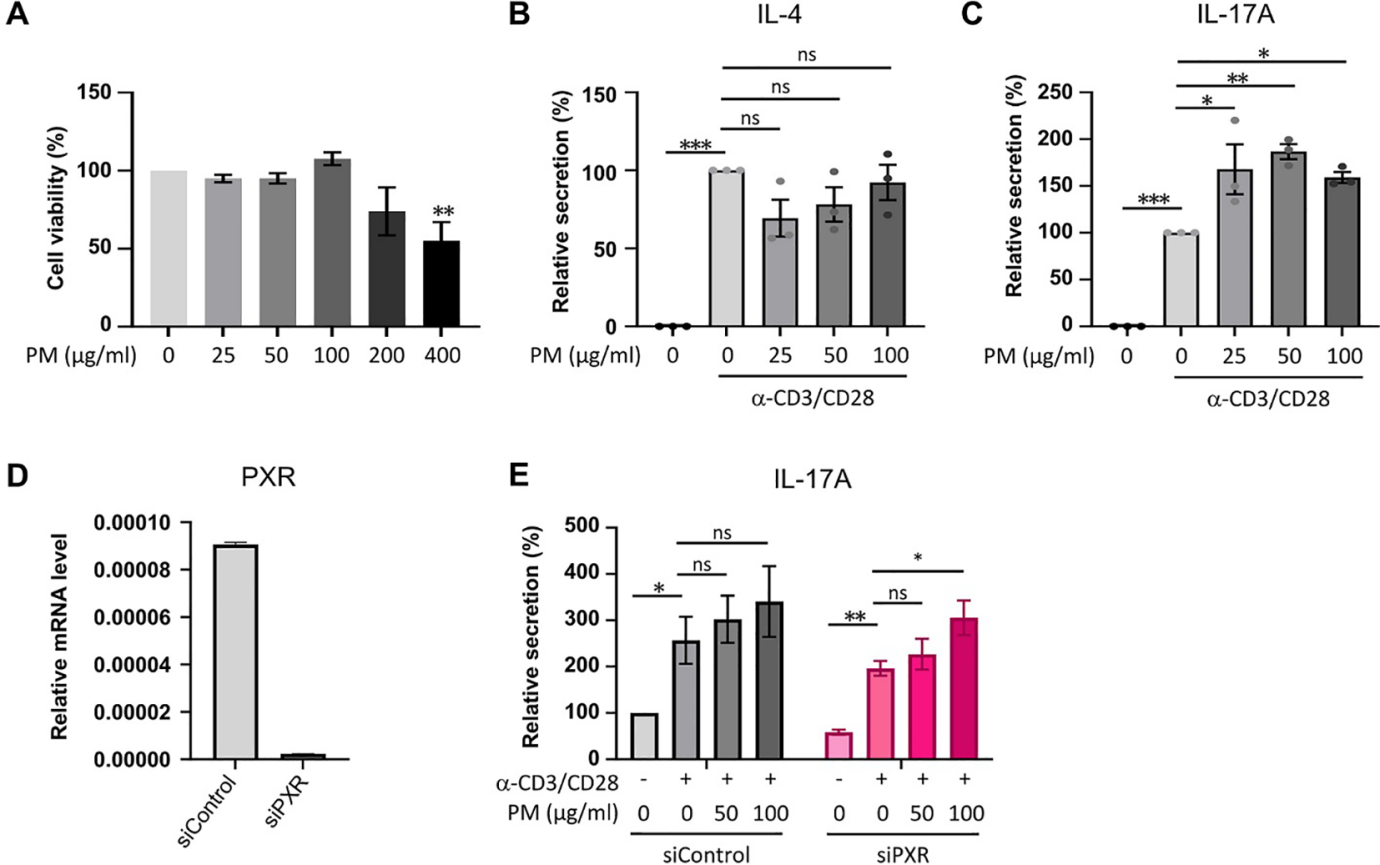
The exposure to PM amplifies IL-17A production in activated CD4+ T cells, with a more pronounced effect observed in PXR knockdown CD4+ T cells. **(A)** Assessment of cell viability in mouse CD4+ T cells exposed to varying concentrations of PM. **(B, C)** Quantification of IL-4 **(B)** and IL-17A **(C)** protein levels in mouse CD4+ T cells following stimulation with anti-CD3/CD28 in the presence of varying concentrations of PM. **(D)** Expression mRNA levels of PXR in control or PXR siRNA transfected mouse CD4+ T cells. **(E)** Evaluation of IL-17A production in mouse CD4+ T cells transfected with control or PXR siRNA, stimulated with anti-CD3/CD28 in the presence of PM (n=5). The relative secretions of IL-17A were normalized to those of the untreated control siRNA-transfected group. Data are representative of two independent experiments and are shown as the mean ± SEM (n = 3-5 mice). ns, nonsignificant; *P <.05; **P <.01; ***P <.001. P-values were obtained employing the unpaired Student’s t-test and one-way ANOVA.

To assess the direct impact of PM on IL-17A production in activated CD4^+^ T cells *in vitro*, we utilized CD4^+^ T cells from mouse splenocytes and subjected them to stimulation with varying concentrations of PM to establish an optimal concentration. After a 24-hour stimulation with 25, 50, or 100 μm/ml of PM, there were no observed cytotoxic effects on mouse CD4^+^ T cells compared to control cells; however, exposure to 200 or 400 μg/ml of PM exhibited cytotoxic effects on the cells relative to control (**Figure 5A**). ELISA analysis revealed a significant enhancement in IL-17A production, but not IL-4, in anti-CD3/CD28-stimulated CD4^+^ T cells when exposed to 25, 50, or 100 μm/ml of PM (**Figures 5B, C**). Furthermore, the knockdown of PXR significantly enhances the production of IL-17A of anti-CD3/CD28-stimulated CD4+ T cells in response to PM exposure. After 100 µg/ml PM treatment in anti-CD3/CD28-stimulated CD4+ T cells, IL-17A production increased by 55.7% in PXR siRNA-transfected group, compared to 32.6% in control siRNA-transfected group (**Figures 5D, E**). These findings suggest that exposure to PM enhances IL-17A production in activated CD4^+^ T cells, and PXR suppresses PM-induced IL-17A production in these cells.

### 3.7 PM-induced PXR activation suppresses the NF-κB signaling pathway

**Figure 6:**
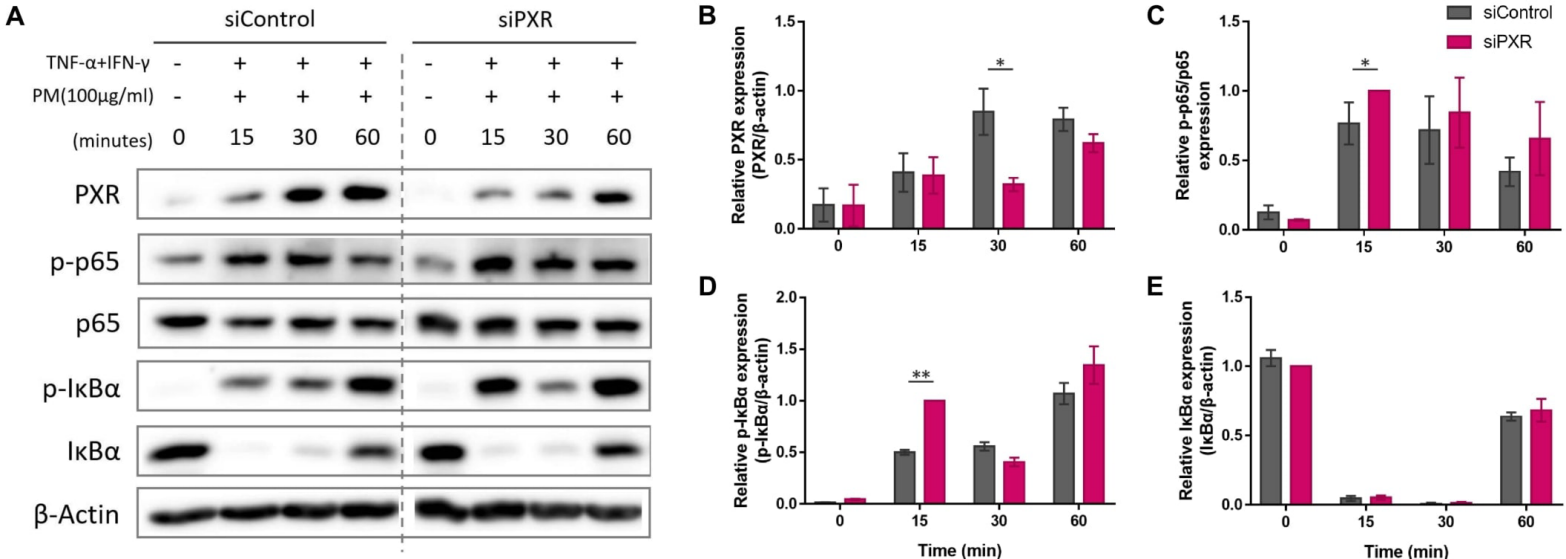
Comparison of the expression of the nuclear factor kappa B (NF-κB) signaling pathway components after particulate matter (PM) treatment between control and pregnane X receptor (PXR) siRNA-transfected atopic dermatitis (AD)-like keratinocytes. **(A, B)** In control siRNA-transfected keratinocytes, PM gradually increased PXR protein levels up to 30 min and remained at a similar level at 60 min. **(A, C)** Control siRNA-transfected keratinocytes exhibited an increase in phosphorylated p65 levels up to 30 min, followed by a decrease at 60 min. In PXR siRNA-transfected keratinocytes, PM induced an earlier and more substantial increase in phosphorylated p65 levels, reaching their peak at 15 min and maintaining slightly lower levels until 60 min. **(A, D)** In control siRNA-transfected keratinocytes, phosphorylated IκBα reached its maximum level at 60 min, whereas in the PXR siRNA-transfected keratinocytes, its peak level occurred earlier, at 15 and 60 min. **(A, E)** IκBα degraded at first and then recovered in both groups. Collectively, the PM-induced activation of PXR might constrain NF-κB signaling, potentially contributing to the reduction of type 17 inflammation caused by PM. Three independent experiments were performed. **(A)** shows representative immunoblot images from these experiments. In **(B–E)**, data from all three experiments are presented as mean ± SEM (n = 3 per group). For panels **(B, D, E)**, the expressions of PXR, phosphorylated IκBα, and IκBα were normalized to β-actin. **(C)** shows the expression ratio of phosphorylated p65 to total p65. *P <.05; **P <.01. P-values were obtained using the unpaired Student’s t test.

**Figure 7:**
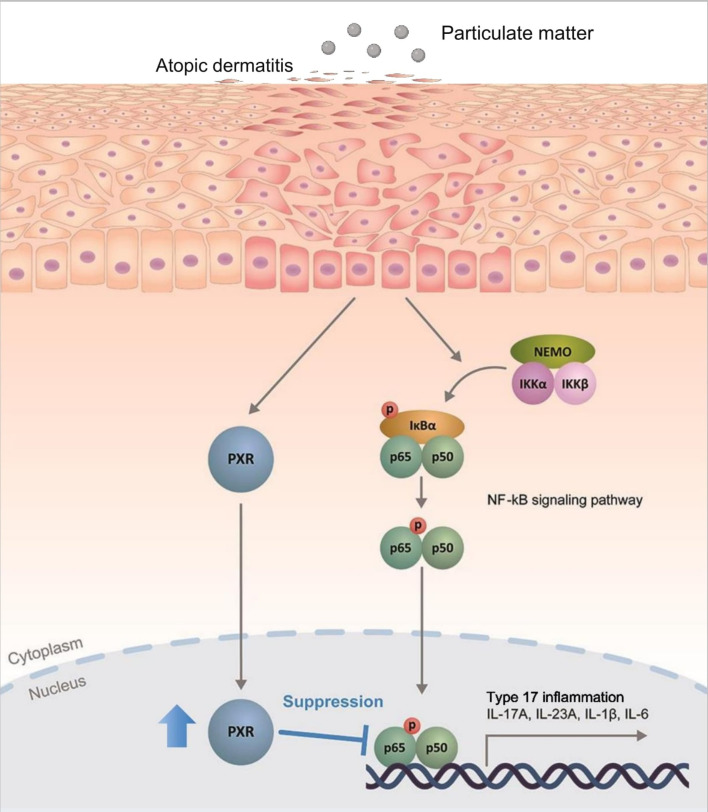
Schematic illustration demonstrating the protective role of pregnane X receptor (PXR) in particulate matter (PM)-exposed atopic dermatitis. PM induces type 17 inflammation through nuclear factor kappa B (NF-κB) signaling pathway in AD. Simultaneously, PM activates PXR and activated PXR suppresses NF-κB signaling pathway, mitigating the PM-induced type 17 inflammation in AD.

Next, we sought to identify the signaling pathway mediated by PXR activation. PXR and NF-κB pathways are known to exert reciprocal repression (15, 49). PM has been shown to activate the NF-kB signaling pathway (2, 6, 17, 32, 50) which promotes Th17 differentiation and IL-17 and IL-23 production (50, 51). We investigated whether the antagonistic relationship between PXR and NF-κB was involved in PM-exposed AD by comparing the activation of NF-κB signaling pathway between control and PXR siRNA-transfected AD-like keratinocytes after PM treatment (**Figure 6**). In control siRNA-transfected keratinocytes, PM gradually increased PXR protein levels up to 30 min, which remained at a similar level at 60 min (**Figures 6A, B**). Additionally, these keratinocytes exhibited an increase in phosphorylated p65 levels up to 30 min, followed by a decrease at 60 min (**Figures 6A, C**). In contrast, in PXR siRNA-transfected keratinocytes, PM induced an earlier and more substantial increase in phosphorylated p65 levels, reaching their peak at 15 min and maintaining slightly lower levels until 60 min (**Figures 6A, C**). In control siRNA-transfected keratinocytes, phosphorylated IκBα reached its maximum level at 60 min, whereas in the PXR siRNA-transfected keratinocytes, its peak level occurred earlier, at 15 and 60 min (**Figures 6A, D**). Phosphorylated p65 levels increased following SPA70 treatment (**Supplementary Figure 6**), which was consistent with the increased phosphorylated p65 levels observed in PXR knockdown keratinocytes. These findings indicate that PM exposure in the context of PXR knockdown resulted in an earlier activation of IκBα, followed by a more potent and earlier activation of p65 in AD-like keratinocytes (**Figure 7**). Conversely, the PM-induced activation of PXR might constrain NF-κB signaling, potentially contributing to the reduction of type 17 inflammation caused by PM (**Figure 7**).

## 4 Discussion

PM can penetrate intact and barrier-disrupted skin through hair follicles and intercellularly in barrier-disrupted skin (52). We previously showed that repeated topical application of PM resulted in neutrophil-dominant dermal inflammation in barrier-disrupted skin (52). Epidemiological studies showed that PM contributes to the aggravation and development of AD (2, 6, 11–14). The underlying mechanisms have been suggested to involve skin barrier impairment, dysfunctional inflammation, microbiome alteration, and oxidative damage (6).

PM induces type 17 inflammation in epithelial and immune cells in the lung (42, 43, 45). In ovalbumin-treated mouse skin and keratinocytes, PM exposure increased transcripts related to Th17 cell differentiation and the IL-17 signaling pathway (17, 48). In the present study, we showed that PM exposure induces robust type 17 inflammation in both *in vivo* and *in vitro* AD-like inflammation models. In addition to type 2 immune responses, type 17 immune response is essential in AD pathogenesis (38). IL-17, the main cytokine in type 17 immune response, regulates type 2 immune response, stimulates epithelial cells and fibroblasts to secrete pro-inflammatory cytokines such as IL-8 and IL-6, and promotes tissue fibrosis (38, 53). IL-17 contributes to the progression of AD to chronic disease (38, 39). We previously showed that the IL-17A/IL-33 circuit amplifies and sustains chronic AD (40). Collectively, our results suggest that PM can accelerate the progression to the chronic phase of AD via the activation of type 17 inflammation. Notably, AD in the Asian population had greater Th17 polarization and psoriasiform features such as parakeratosis and increased neutrophils compared to AD in European-Americans (54–56), and PM exposure may aggravate the psoriasiform features of AD in Asian people. Coincidently, according to the World Air Quality Report 2021, 16 out of the 20 most polluted countries in the world are located in Asia. This implies that severe air pollution in Asia may affect the unique phenotype of AD in the Asian population.

Recent studies have shown that PXR signaling was activated in the skin of patients with AD (18) and downstream genes of human PXR encoding uridine-5’-diphospho-glucuronosyltransferases (UGTs) were increased in AD and PM-exposed AD (16, 17), but the role of PXR remained unclear. We observed that PM activated PXR in AD-like inflammation models both *in vivo* and *in vitro*. The transcriptional regulatory mechanisms that govern PXR gene expression are complex and not fully understood. Ets-1 and peroxisome proliferator-activated receptor alpha (PPARα) have been identified as key regulators, binding to proximal promoter sites of PXR and modulating its expression (57, 58). Interestingly both Ets-1 and PPARα appear to be implicated in AD pathogenesis. Mice deficient in either factor exhibit AD-like features, and their expression is diminished in the skin of AD patients compared to healthy controls (59, 60). These findings suggest a potential link between PXR regulation and AD development, warranting further investigation. In our study, PXR knockdown exacerbated PM-induced type 17 inflammation in AD-like keratinocytes, as well as IL-17A production in CD4^+^ T cells. Furthermore, the use of a PXR agonist, rifampicin, reduced this inflammation, indicating that PXR activation in PM-exposed AD can protect against PM-induced type 17 inflammation. In contrast, overexpression of PXR may induce pro-inflammatory effects and produce AD-like inflammation as transgenic mice constitutively expressing human PXR in basal keratinocytes (K14-VPPXR mice) exhibited AD-like features such as skin barrier impairment, increased serum IgE, and increased Th2/Th17 skin immune responses (18). This contrasting role of PXR might be context- and ligand-dependent (15). It is possible that the persistent and basal state where PXR activation occurs may affect its pro- or anti-inflammatory role. Similarly, the activation of AHR by pollutants induces inflammation in exposed skin, but therapeutic AHR-modulating agents such as tapinarof show anti-inflammatory effects in inflammatory disorders such as AD and psoriasis (15, 61–63).

The NF-κB signaling pathway, implicated in the progression and maintenance of AD, is an important molecular target in the treatment of AD (64). NF-κB promotes the production of IL-17 and other pro-inflammatory cytokines such as IL-6 and IL-1. Meanwhile, IL-17 activates NF-κB in a feed-forward manner and stimulates the production of inflammatory mediators and keratinocyte proliferation (50, 51). PXR and NF-κB pathways are known to reciprocally suppress each other, in a context-dependent manner (15, 49). Consistently, our results suggest that PXR activation, induced by PM exposure, mediates the inhibition of NF-kB signaling pathway in AD, which might contribute to reducing type 17 inflammation and the associated exacerbation of AD.

This study has several limitations. First, our PM treatment may not fully mimic real-life exposure. Quantifying PM accumulation on the skin is challenging due to heterogeneous nature of PM, varying concentrations depending on geographical and meteorological factors, and differences in individual skin conditions. In the present study, we selected PM concentrations based on previous experimental studies, aiming to reflect realistic exposures. We employed PBS as the vehicle for PM suspension to minimize potential cytotoxicity and mouse skin irritation. Although many PM components exhibit limited water solubility, we tried to achieve uniform dispersion of PM in PBS through vigorous sonication, to ensure consistent exposure conditions while maintaining cellular and tissue viability. However, further research is needed to validate these conditions and ensure they accurately represent real-world exposure. Additionally, future studies may include comparative analyses using different vehicles, including organic solvents, to evaluate their effects on PM activity and cellular responses. Second, the manipulation of PXR expression and activation was performed only *in vitro*, necessitating further confirmation through *in vivo* experiments. To further validate and extend our results, future studies should employ PXR knockout mouse models and administer PXR agonist treatments in mice with AD-like conditions.

This study provides evidence that PM intensifies type 17 inflammation in AD, both *in vivo* and *in vitro*. Furthermore, it uncovers a previously unappreciated role of PXR, which is activated by PM and plays a crucial role in reducing PM-induced type 17 inflammation through suppressing the NF-kB signaling pathway. Consequently, targeting PXR may represent a potential therapeutic approach to mitigate the progression toward chronic or psoriasiform AD triggered by PM exposure. Given the complexity of the immune responses of AD, further investigations in varied *in vivo* and *in vitro* models is necessary to confirm the role of PXR in PM-exposed AD. Moreover, delving into the potential protective effect of PXR agonists such as rifampicin against air pollution in human subjects warrants further investigation.

## Data Availability

The original contributions presented in the study are included in the article/**Supplementary Material**. Further inquiries can be directed to the corresponding authors.
