# Supplementary material for: Pregnane X receptor reduces particulate matter-induced type 17 inflammation in atopic dermatitis
Source: Front Immunol. 2024 Sep 27;15:1415350. doi: 10.3389/fimmu.2024.1415350 (PMC11467722; doi:10.3389/fimmu.2024.1415350)

***Supplementary Materials***

**Supplementary Methods
Supplementary Table
Supplementary Figures (1-10)
Supplementary Data (excel file 1-12)**

**Pregnane X receptor reduces particulate matter-induced type 17 inflammation in atopic dermatitis**

**Ji Su Lee ^1,2†^, Youngae Lee^2,3,4†^, Sunhyae Jang^2,4,5^, Jang-Hee Oh^1,3,4^, Dong Hun Lee^1,2,3,4^*, Soyun Cho, M.D^1,4,6^***

^1^Department of Dermatology, Seoul National University College of Medicine, Seoul, Republic of Korea ^2^Department of Dermatology, Seoul National University Hospital, Seoul, Republic of Korea ^3^Laboratory of Cutaneous Aging Research, Biomedical Research Institute, Seoul National University Hospital, Seoul, Republic of Korea ^4^Institute of Human-Environment Interface Biology, Medical Research Center, Seoul National University, Seoul, Republic of Korea ^5^Laboratory of Cutaneous Aging and Hair Research, Clinical Research Institute, Seoul National University Hospital, Seoul, Republic of Korea ^6^Department of Dermatology, Seoul Metropolitan Government – Seoul National University (SMG-SNU) Boramae Medical Center, Seoul, Republic of Korea

†Ji Su Lee and Youngae Lee contributed equally to this work and share first authorship.
*Dong Hun Lee and Soyun Cho contributed equally to this work and share corresponding authorship.

**Correspondence:**

Dong Hun Lee, M.D., Ph.D.
Department of Dermatology, Seoul National University Hospital, 101 Daehak-ro, Jongno-gu, Seoul 03080, Republic of Korea
Tel: +82-2-2072-2418, Fax: +82-2-742-7344, E-mail: ivymed27@snu.ac.kr

Soyun Cho, M.D., Ph.D.
Department of Dermatology, Seoul Metropolitan Government – Seoul National University (SMG-SNU) Boramae Medical Center, 20, Boramae-ro 5-gil, Dongjak-gu, Seoul 07061, Republic of Korea
Tel: +82-2-870-2381, Fax: +82-2-870-3866, E-mail: sycho@snu.ac.kr

**Supplementary Methods**

***Ethical approval***

The animal experimental protocol was approved by the Seoul National University Hospital Institutional Animal Care and Use Committee (No.19-0090-S1A0). All experiments were performed in accordance with the approved experimental protocol.

***Dermatitis scoring***

On Day 17, before sacrificing, the dermatitis score was determined according to the criteria previously described, with slight modifications. The score was defined as the sum of each score graded as 0 (none), 1 (mild), 2 (moderate), or 3 (severe) for four signs: erythema, edema/papulation, erosion/excoriation, and dryness/scaling.

***Histopathological assessment***

On Day 17, mice were anesthetized and sacrificed to obtain a sample of dorsal skin and serum. Excised dorsal skin tissues were fixed in 4% formalin and embedded in paraffin. Then, 4 µm thick sections were prepared and stained with hematoxylin and eosin (H&E). Epidermal thickness (distance between the granular and basal layers of the epidermis) and dermal thickness (distance between the bottom of the basal layer of the epidermis and the top of the subcutaneous layer) were quantified by measuring at three randomly selected spots in H&E-stained slides of each mouse skin.

***PM-induced cell cytotoxicity***

At 24 h after 0, 25, 50, 100, 200, and 400 μg/ml of PM treatment, Cell Counting Kit-8 (WST-8) solution was added to each well with fresh growth media, and the plates were incubated at 37 °C for 60 min. Then, absorbance was measured at 460 nm using a microplate reader. Relative viability was calculated from the ratio of absorbance in each group. The concentration of PM for treatment (100 μg/ml) was selected as the highest concentration without cytotoxicity.

***siRNA transfection***

PXR expression was knocked down using small interfering RNAs (siRNAs) at the starvation step, 1 day before co-stimulation with TNF-α and IFN-γ. Negative control siRNA (# 4390843) and PXR siRNA (#4390818, s16911) were purchased from Invitrogen (Carlsbad, CA, USA). Control siRNA or PXR siRNA were each transfected into HaCaT cells using Metafectene^®^ Pro (Biontex, Martinsried/Planegg, Germany) following the manufacturer’s protocol and incubated for 24 h. After transfection, TNF-α (2 ng/mL) and IFN-γ (10 ng/mL) co-stimulation and PM treatment were performed as described above. Mouse CD4^+^ T cells (1x10^6^) were transfected for 24h with either negative control siRNA or mouse PXR siRNA (1500 nM) via electroporation using a NEPA21 electroporator (NEPA GENE Co., Ltd., Japan) following the manufacturer’s protocols. The siRNAs were purchased from Bioneer Co. (Daejeon, Korea). Consequently, 24 h post-transfection, the cells were used in subsequent experiments.

***Rifampicin, SPA70 and CH223191 treatment***

Rifampicin, a potent human PXR agonist, was treated at the starvation step, 1 day before co-stimulation with TNF-α and IFN-γ. Rifampicin powder was purchased from Sigma-Aldrich (#3501, St Louis, MO, USA) and dissolved in <0.1% of dimethyl sulfoxide (DMSO). The negative control was DMSO alone (0.05%). After rifampicin treatment, TNF-α (2 ng/mL) and IFN-γ (10 ng/mL) co-stimulation and PM treatment were performed as described above. SPA70, a human PXR antagonist and CH-223191, a human AHR antagonist, were administered to keratinocytes 90 min before the TNF-α, IFN-γ, and PM treatments. SPA70 and CH223191 were purchased from Sigma-Aldrich.

**Quantitative real-time reverse transcription polymerase chain reaction (PCR)**

Mouse skin tissues and cells were lysed in RNAiso Plus (Takara Bio Inc., Shiga, Japan), and total RNA was isolated following the manufacturer's protocol. The total RNA (1 μg) was used for cDNA synthesis using the RevertAid First Strand cDNA Synthesis Kit (Thermo Fisher Scientific) and quantified with SYBR Premix Ex Taq (Takara Bio) with ROX using a 7500 Real-time PCR system (Applied Biosystems, Foster City, CA, USA) and respective primer pairs. The expression was normalized to the expression level of mouse/human 36B4 mRNA.

<Sequences of polymerase chain reaction (PCR) primers>

| **Gene** | **Primers (5′ - 3′)** | |
| --- | --- | --- |
|  | **Forward** | **Reverse** |
| m36B4 | TGCCACACTCCATCATCAAT | CGAAGAGACCGAATCCCATA |
| mIL-4 | CAGCTAGTTGTCATCCTGGTCTTC | GCCGATGATGTCTCTCAAGTGA |
| mIL-13 | GCTTATTGAGGAGCTGAGCAACA | GCCAGGTCCACACTCCATA |
| mIL-17A | GCAATGAAGACCCTGATAGATATCC | TTCATGTGGTGGTCCAGCTTT |
| mIL-23A | GCTGTGCCTAGGAGTAGCAG | AGTCCTTGTGGGTCACAACC |
| mIL-1β | TGTAATGAAAGACGGCACACC | TCTTCTTTGGGTATTGCTTGG |
| mIL-6 | GCTACCAAACTGGATATAATCAGG | CCAGGTAGCTATGGTACTCCAGAA |
| mPXR | GGTTTTTCAGAAGGGCCATGA | CGGGTGATCTCGCAGGTT |
| mCYP1B1 | GCCACTATTACGGACATCTTCGG | ACAACCTGGTCCAACTCAGCCT |
| mCYP3A11 | GACAAACAAGCAGGGATGGAC | CCAAGCTGATTGCTAGGAGCA |
| h36B4 | AGATGCAGCAGATCCGCAT | ATATGAGGCAGCAGTTTCACCAG |
| hIL-17A | TCCCACGAAATCCAGGATGC | GGATGTTCAGGTTGACCATCAC |
| hIL-23A | CAGCAACCCTGAGTCCCTAA | TCAACATATGCAGGTCCCACT |
| hIL-1β | AGCTCGCCAGTGAAATGATG | TGGTGGTCGGAGATTCGTAG |
| hIL-6 | CCACTCACCTCTTCAGAACG | CCACTCACCTCTTCAGAACG |
| hPXR | GCTGTCCTACTGCTTGGAAGAC | CTGCATCAGCACATACTCCTCC |
| hUGT1A1 | CCTTGCCTCAGAATTCCTTC | ATTGATCCCAAAGAGAAAACCAC |

**Enzyme-linked immunosorbent assay (ELISA)**

In the *in vivo* experiments, harvested mouse dorsal skin tissues were lysed using Tissue Lyser II (Qiagen, Venlo, Limburg, Netherlands) within the cell lysis buffer of the Bio-Plex Cell Lysis Kit (Bio-Rad Laboratories, Hercules, CA, USA). Extracted proteins were quantified using the bicinchoninic acid protein assay. The samples were diluted (1 mg/mL) with an equal volume of cell lysis buffer. In the *in vitro* experiments, supernatants were collected from cells after 24 h of PM treatment. Protein concentrations of IL-17A, IL-1β, and IL-6 in tissue lysates and supernatants were quantified using Bio-Plex Pro Mouse Cytokine & Chemokine Assays on Bio-Plex^®^ multiplex system (Bio-Rad Laboratories). To measure the protein level of IL-23A in mouse tissue lysates, we used mouse IL-23 DuoSet ELISA kits (DY1887, R&D Systems), per manufacturer’s instructions. Serum IgE levels of mice were measured using mouse IgE ELISA kits (ab157718, Abcam, Cambridge, UK).

**Western blotting**

In the *in vivo* experiments, total protein was extracted from harvested mouse dorsal skin tissue. In the *in vitro* experiments, cells were lysed using RIPA buffer (Sigma-Aldrich) mixed with a protease inhibitor (Roche Applied Science, Penzberg, Germany) and phosphatase inhibitor cocktail (Sigma-Aldrich). The Bradford assay was used to quantify the extracted total protein. Equal amounts of protein (40 μg in *in vivo* experiments; 30 μg in *in vitro* experiments) were separated by 8% SDS-PAGE and transferred onto polyvinylidene fluoride membranes (Sigma-Aldrich). The membranes were probed with antibodies against mouse PXR (ab192579, 1:1000; Abcam), human PXR (PA5-115667, 1:1000, Invitrogen), p-p65 (#3031, 1:1000, Cell Signaling Technology, Beverly, MA, USA), p65 (#8242, 1:1000, Cell signaling Technology), p-IκBα (#2859, 1:1000, Cell signaling Technology), IκBα (#9242, 1:1000, Cell signaling Technology), and β-actin (sc-47778, 1:1000; Santa Cruz Biotechnology, Santa Cruz, CA, USA). Immunoreactive proteins were visualized using the enhanced chemiluminescence detection system (Biomax Co. Ltd., Seoul, Republic of Korea). Signal intensity was measured using ImageJ software (NIH, Bethesda, MD, USA).

**Luciferase reporter assay**

Luciferase activity of human PXR was measured 24 h after treatment with TNF-α, IFN-γ, and PM, using a human PXR reporter assay kit (INDIGO Biosciences, State College, PA, USA) according to the manufacturer’s protocol.

**Supplementary Table**

**Supplementary Table 1.** Composition of standard reference material 2786

|  |  | **Mass fraction** |
| --- | --- | --- |
| **Polycyclic aromatic hydrocarbons (PAHs)** | Fluorene | 0.195 ± 0.014 (mg/kg) |
|  | Fluoranthene | 10.28 ± 0.36 (mg/kg) |
|  | Pyrene | 8.01 ± 0.22 (mg/kg) |
|  | Benzo[ghi]fluoranthene | 3.158 ± 0.098 (mg/kg) |
|  | Benzo[c]phenanthrene | 1.597 ± 0.052 (mg/kg) |
|  | Benz[a]anthracene | 4.82 ± 0.17 (mg/kg) |
|  | Chrysene | 6.82 ± 0.53 (mg/kg) |
|  | Triphenylene | 1.794 ± 0.041 (mg/kg) |
|  | Benzo[b]fluoranthene | 7.51 ± 0.36 (mg/kg) |
|  | Benzo[j]fluoranthene | 4.37 ± 0.32 (mg/kg) |
|  | Benzo[k]fluoranthene | 3.48 ± 0.32 (mg/kg) |
|  | Benzo[a]fluoranthene | 0.898 ± 0.037 (mg/kg) |
|  | Benzo[e]pyrene | 4.77 ± 0.28 (mg/kg) |
|  | Benzo[a]pyrene | 3.70 ± 0.13 (mg/kg) |
|  | Perylene | 0.769 ± 0.020 (mg/kg) |
|  | Benzo[ghi]perylene | 5.60 ± 0.41 (mg/kg) |
|  | Indeno[1,2,3-cd]pyrene | 4.87 ± 0.36 (mg/kg) |
|  | Dibenz[a,c]anthracene | 0.509 ± 0.052 (mg/kg) |
|  | Dibenz[a,j]anthracene | 0.610 ± 0.015 (mg/kg) |
|  | Dibenz[a,h]anthracene | 0.717 ± 0.029 (mg/kg) |
|  | Benzo[b]chrysene | 0.662 ± 0.022 (mg/kg) |
|  | Picene | 1.242 ± 0.031 (mg/kg) |
|  | Coronene | 2.156 ± 0.087 (mg/kg) |
|  | Dibenzo[b,k]fluoranthene | 1.013 ± 0.082 (mg/kg) |
|  | Dibenzo[a,e]pyrene | 0.812 ± 0.081 (mg/kg) |
| **Nitro-substituted PAHs**  **(nitro-PAHs)** | 1-Nitropyrene | 85.9 ± 5.5 (μg/kg) |
|  | 2-Nitrofluoranthene | 330 ± 37 (μg/kg) |
| **Polybrominated diphenyl ether (PBDE) congeners** | PBDE 99 (2,2',4,4',5-Pentabromodiphenyl ether) | 7.60 ± 0.03 (μg/kg) |
|  | PBDE 209 (Decabromodiphenyl ether) | 243 ± 14 (μg/kg) |
| **Inorganic constituents** | Mercury | 5.32 ± 0.14 (mg/kg) |
|  | Aluminum | 33480 ± 700 (mg/kg) |
|  | Cadmium | 4.34 ± 0.07 (mg/kg) |
|  | Chromium | 462.2 ± 1.5 (mg/kg) |
|  | Copper | 847 ± 13 (mg/kg) |
|  | Lead | 286 ± 3 (mg/kg) |
|  | Manganese | 780 ± 39 (mg/kg) |
|  | Vanadium | 85.5 ± 6.5 (mg/kg) |

**Supplementary Figures**

Supplementary Figure 1. Change in the expression of inflammatory cytokines after atopic dermatitis (AD) induction and additional particulate matter (PM) treatment in mice. (A, B) mRNA and (C, D) protein levels of IL-1β and IL-6 increased after AD induction and showed an incremental tendency after additional PM treatment.

Data are representative of two independent experiments and are shown as the mean ± SEM (n = 5 mice in each group). The mRNA data were normalized to the AD group. *P < .05; **P < .01; ***P < .001. P-values were obtained by the unpaired Student’s t test and one-way ANOVA.


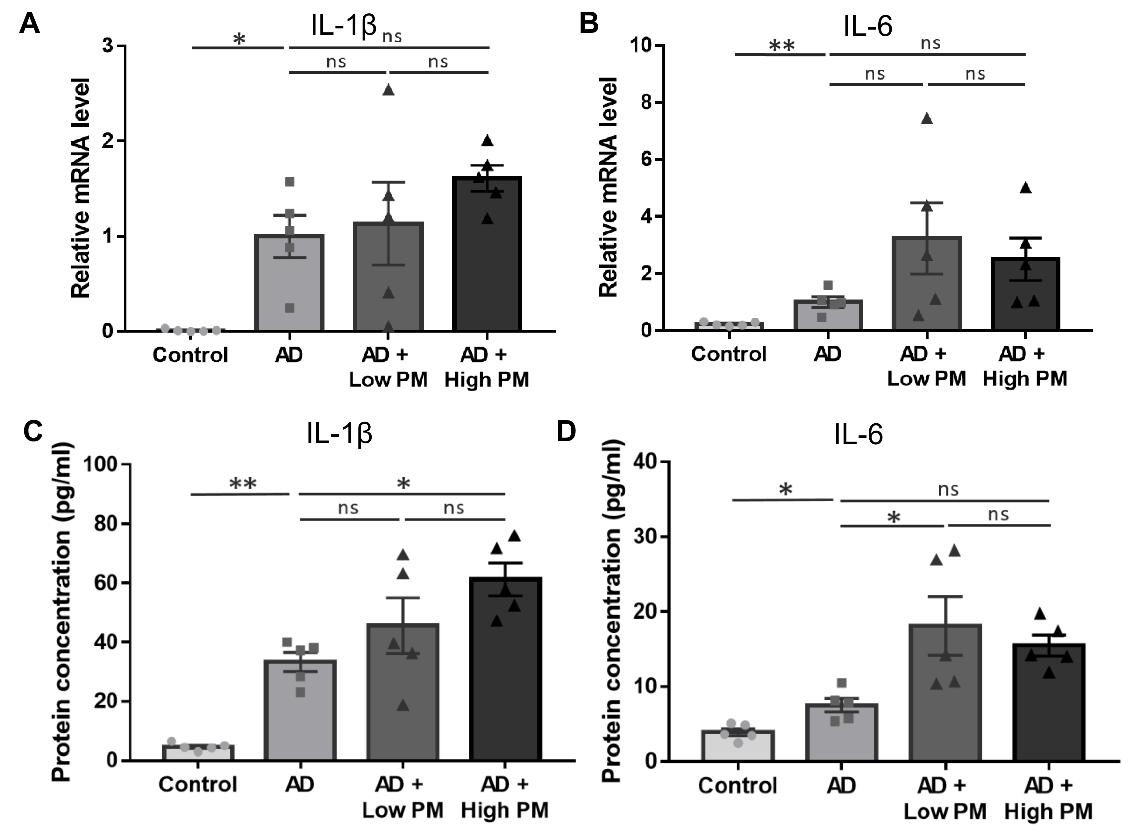


Supplementary Figure 2. Luciferase activity assays demonstrating pregnane X receptor (PXR) activation.

(A) Particulate matter (PM) treatment significantly enhances PXR luciferase activity compared to TNF-α and IFN-γ treatment alone. (B) Rifampicin, a known human PXR agonist, significantly increases PXR luciferase activity compared to the vehicle control.

Data are representative of three independent experiments and are shown as the mean ± SEM (n = 3 in each group). The luciferase activities were normalized to the untreated group. *P < .05; **P < .01. P-values were obtained using the unpaired Student’s t test and one-way ANOVA.


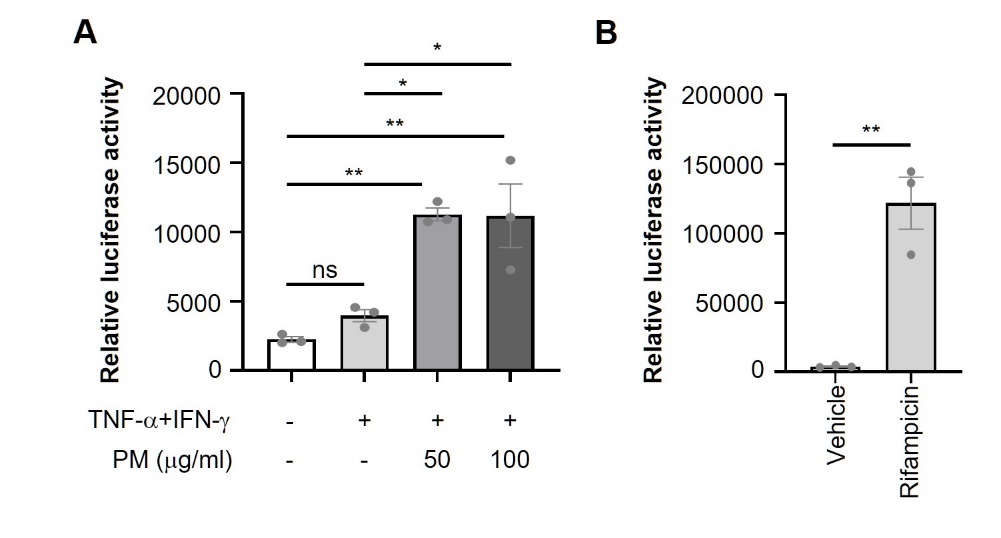


Supplementary Figure 3. Influence of SPA70 (human pregnane X receptor (PXR) antagonist) treatment on particulate matter (PM)-induced inflammation in atopic dermatitis (AD)-like keratinocytes.

(A-C) The mRNA levels of IL-1β, IL-6, and IL-23A increased in PM-treated AD-like keratinocytes. SPA70 treatment further increased the mRNA levels of these cytokines, with the differences being statistically significant for IL-1β, IL-6, and IL-23A.

Data represent three independent experiments and are shown as the mean ± SEM (n = 3 per group). The mRNA data were normalized to TNF-α/IFN-γ/PM-treated keratinocytes. ns, not significant. *P < .05; **P < .01; ***P < .001. P-values were determined using unpaired Student’s t test and one-way ANOVA.


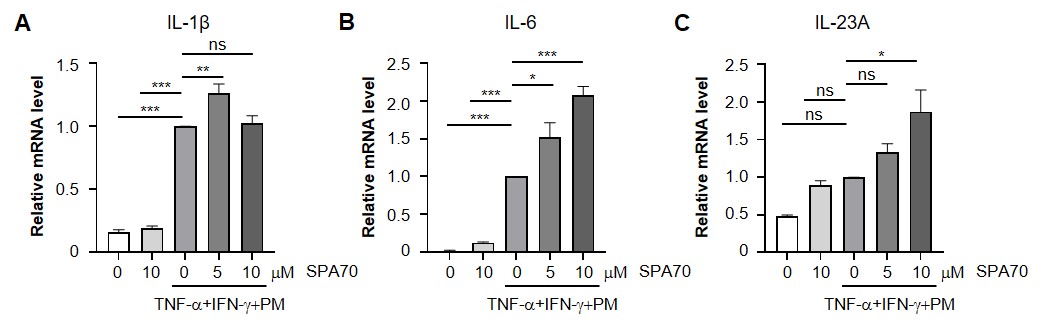


Supplementary Figure 4. Effect of CH223191 (aryl hydrocarbon receptor (AHR) antagonist) treatment on particulate matter (PM)-induced inflammation in atopic dermatitis (AD).

(A-C) The mRNA levels of IL-1β, IL-6, and IL-23A increased in PM-treated AD-like keratinocytes. Treatment with CH223191 resulted in a significant decrease in the expression levels of these cytokines.

Data are representative of three independent experiments and are shown as mean ± SEM (n = 3 per group). The mRNA data were normalized to TNF-α/IFN-γ/PM-treated keratinocytes. ns, not significant. *P < .05; ***P < .001. P-values were determined using unpaired Student’s t test and one-way ANOVA.


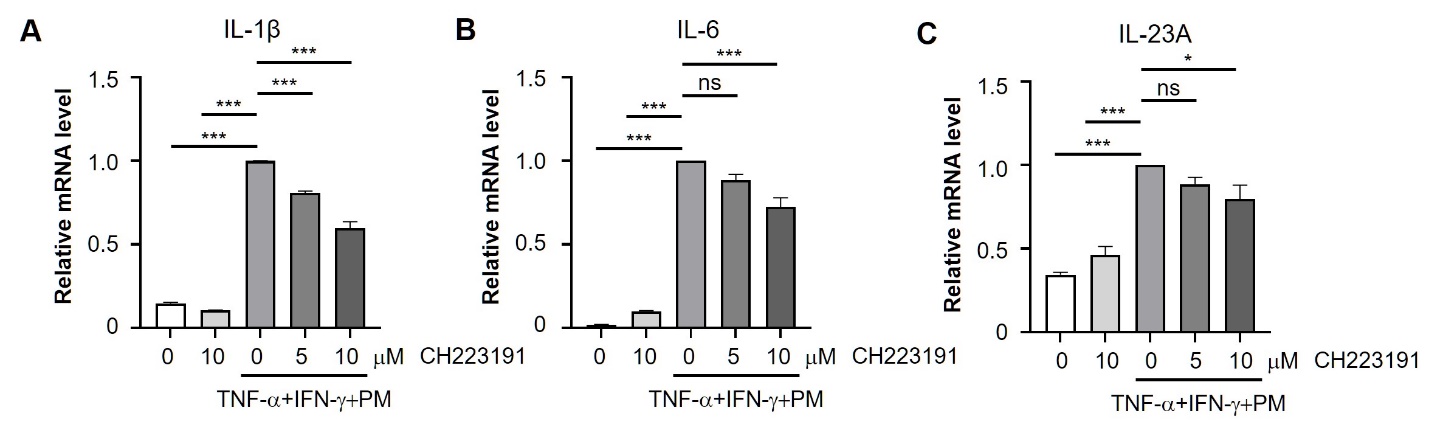


Supplementary Figure 5. Comparison of rifampicin (human pregnane X receptor (PXR) agonist) treatment effects on particulate matter (PM)-induced inflammation in atopic dermatitis (AD) between control and PXR siRNA-transfected keratinocytes. (A, B) Both control and PXR siRNA-transfected keratinocytes showed increased mRNA levels of IL-1β and IL-6 after AD induction and PM treatment (100 µg/ml). Rifampicin treatment (100 µM) decreased these elevated expressions. In the AD-induced, PM and rifampicin-treated groups, the expressions of IL-1β and IL-6 were significantly higher in PXR siRNA-transfected keratinocytes compared to control siRNA-transfected keratinocytes.

Data represent three independent experiments and are shown as the mean ± SEM (n = 3 per group). **P < .01; ***P < .001. P-values were determined using unpaired Student’s t test and one-way ANOVA.
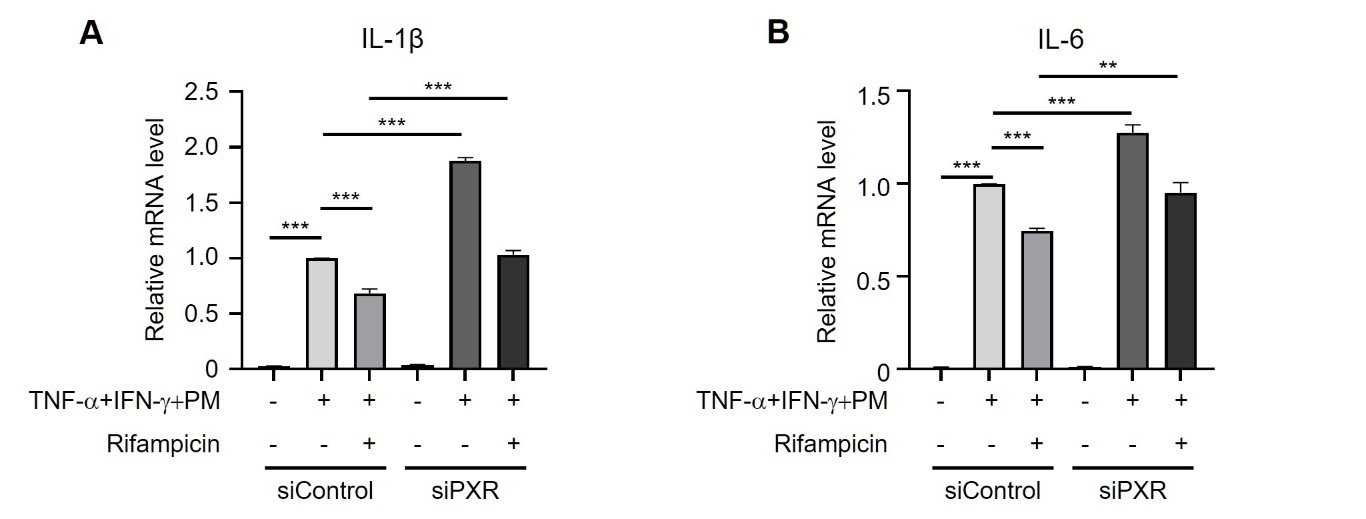


Supplementary Figure 6. Comparison of nuclear factor kappa B (NF-κB) expression between SPA70 (human PXR antagonist) and control (DMSO) in particulate matter (PM)-treated atopic dermatitis (AD)-like keratinocytes.

(A and B) SPA70 treatment significantly increased phosphorylated p65 levels at 30 and 120 minutes compared to control. (A) Representative immunoblot images from two independent experiments. (B) Quantification of phosphorylated p65 levels.

Data from two independent experiments are shown as mean ± SEM. *P < .05. P-values were determined using unpaired Student’s t test.


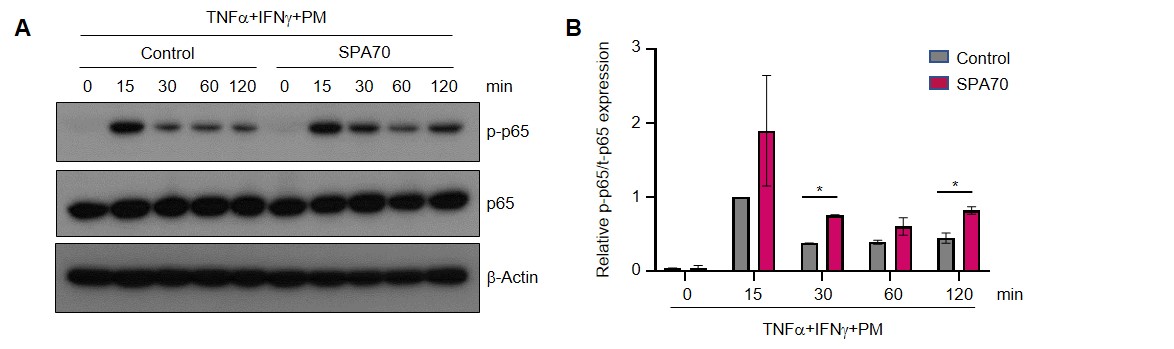


Supplementary Figure 7. Original images for western blot image of figure 2K


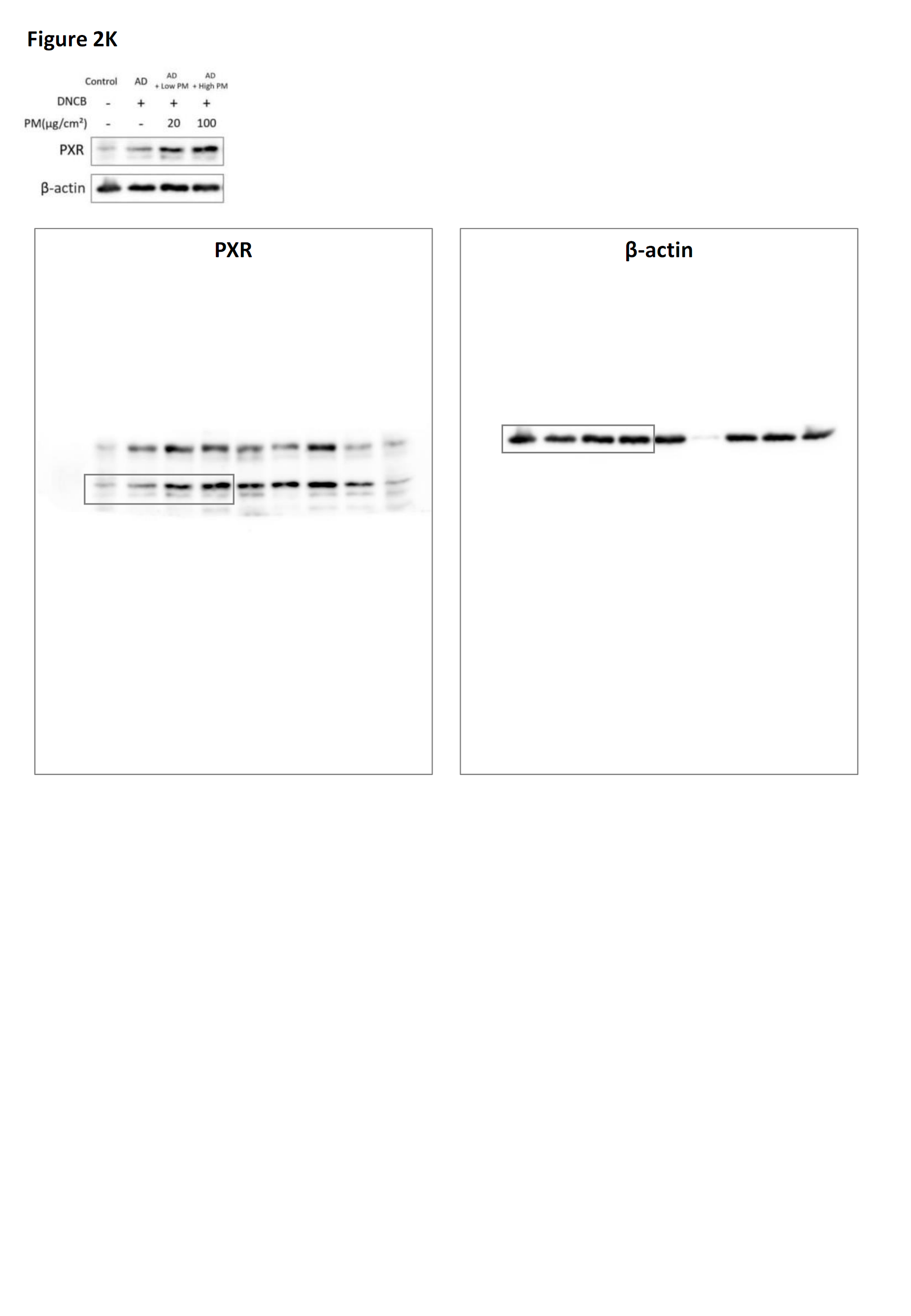


Supplementary Figure 8. Original images for western blot image of figure 3K


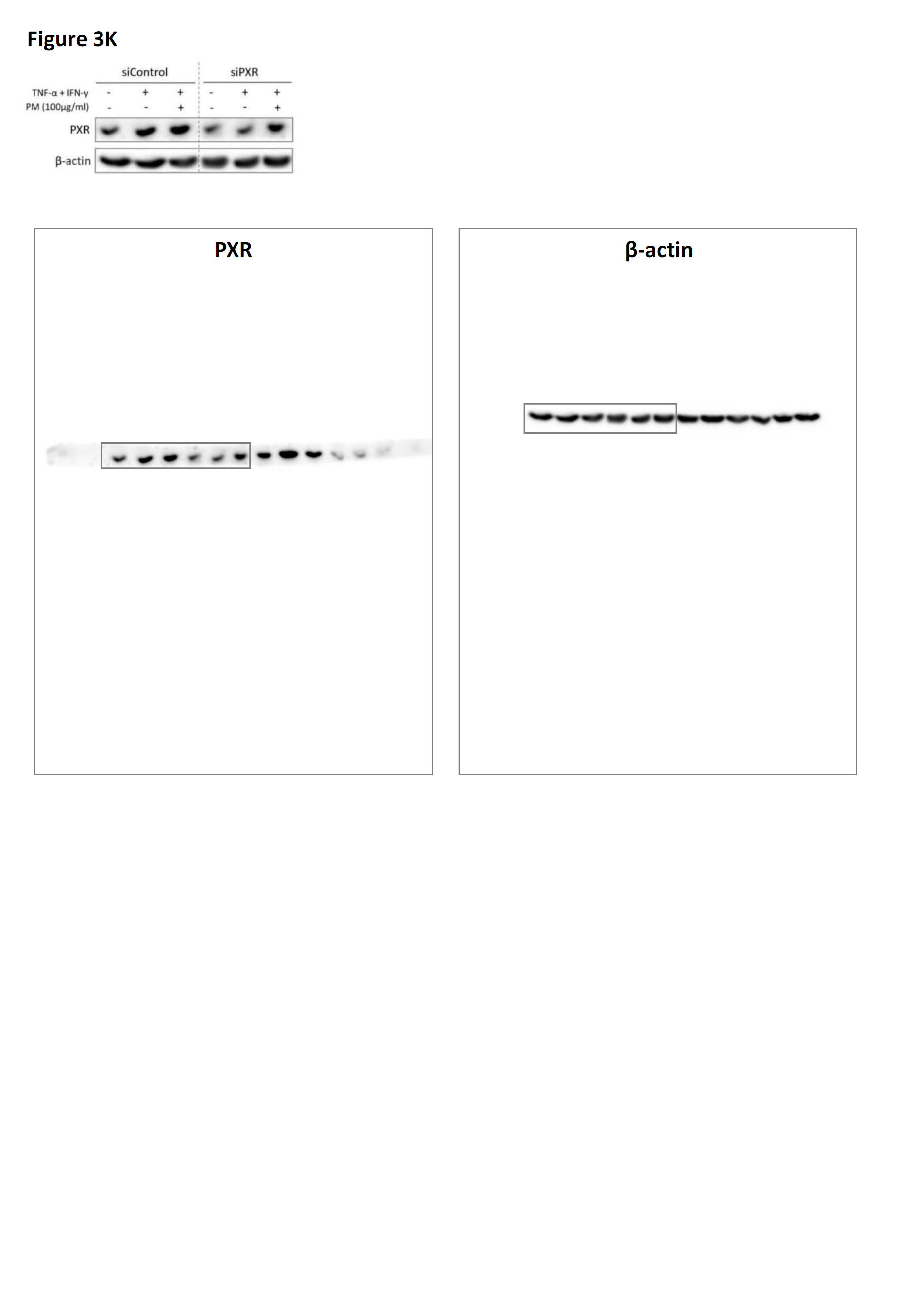


Supplementary Figure 9. Original images for western blot image of figure 6A


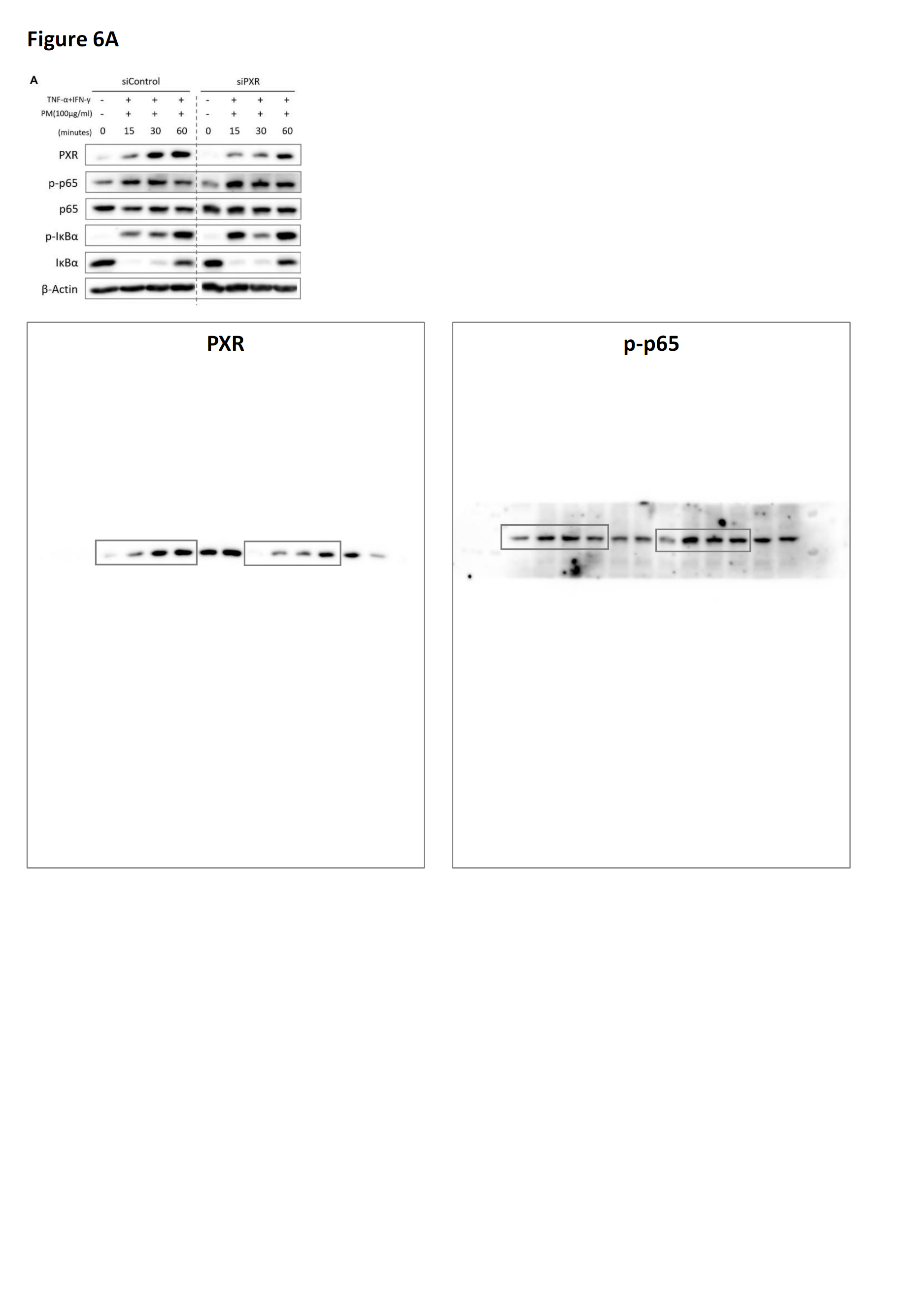


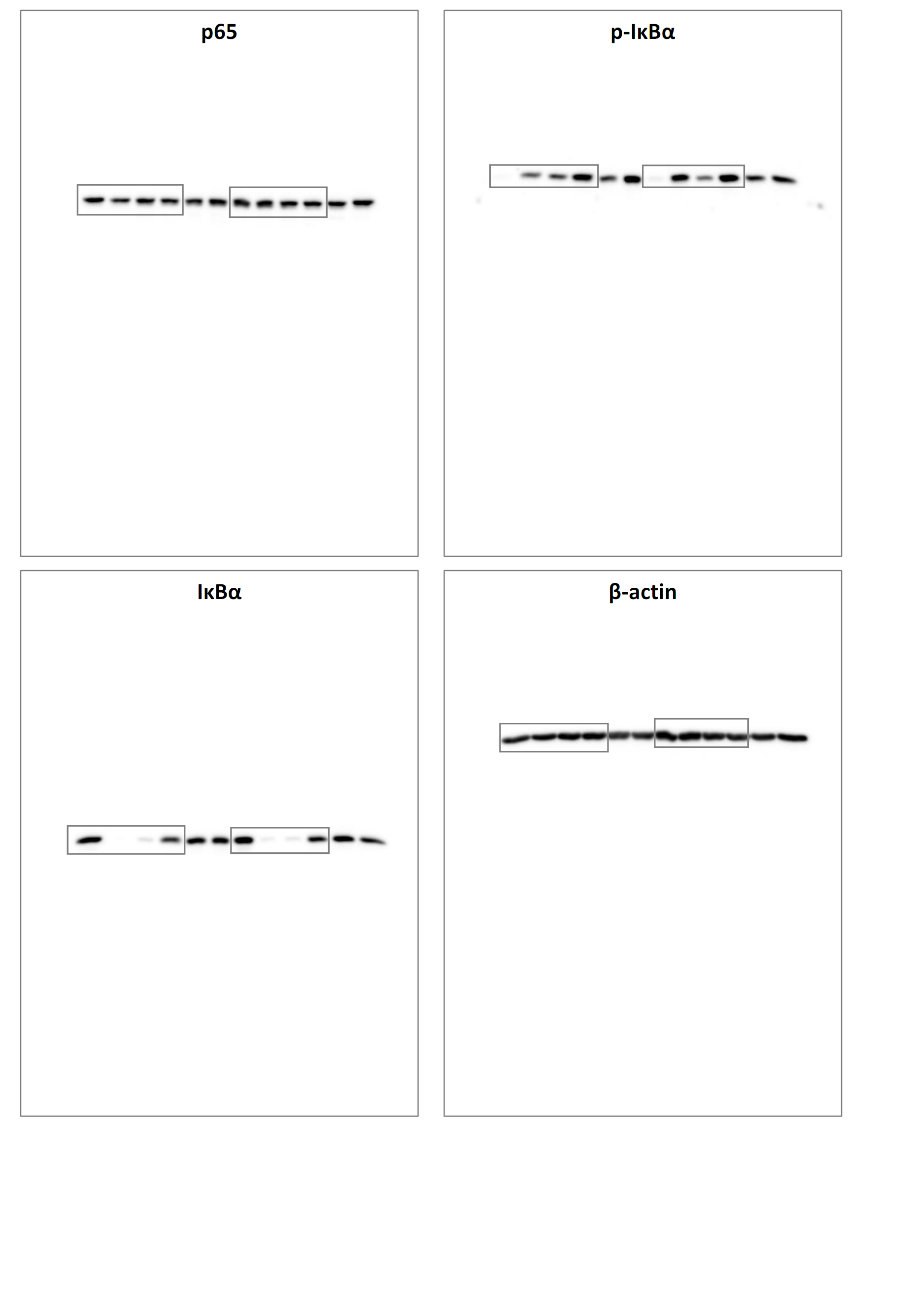


**Supplementary Figure 10. Original images for western blot image of supplementary figure 6**


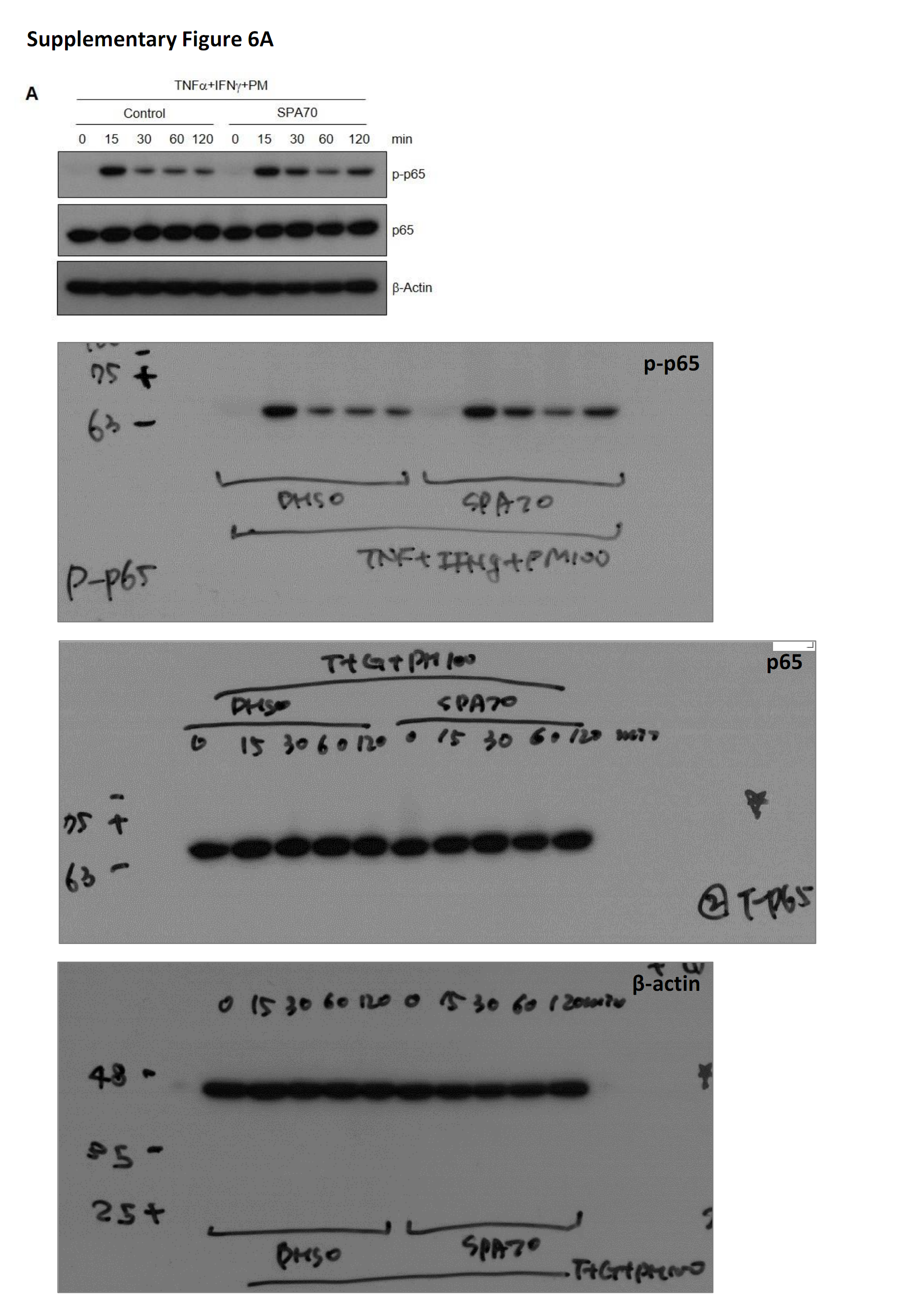

Supplement: Supplementary file 1 [file DataSheet1.zip › Supplementary Methods, Table, and Figures.docx]
